# A molecular survey of Australian and North American termite genera indicates that vertical inheritance is the primary force shaping termite gut microbiomes

**DOI:** 10.1186/s40168-015-0067-8

**Published:** 2015-02-25

**Authors:** Nurdyana Abdul Rahman, Donovan H Parks, Dana L Willner, Anna L Engelbrektson, Shana K Goffredi, Falk Warnecke, Rudolf H Scheffrahn, Philip Hugenholtz

**Affiliations:** Australian Centre for Ecogenomics, School of Chemistry and Molecular Biosciences, The University of Queensland, St Lucia, Brisbane, Queensland Australia; Current address: Department of Statistics, University of Illinois Urbana-Champaign, Champaign, IL USA; DOE Joint Genome Institute, Walnut Creek, CA USA; Current address: Energy Biosciences Institute, University of California, Berkeley, CA USA; Biology Department, Occidental College, Los Angeles, CA USA; Jena School for Microbial Communication (JSMC) and Microbial Ecology Group, Friedrich Schiller University Jena, Jena, Germany; Fort Lauderdale Research and Education Center, University of Florida, Davie, FL USA

## Abstract

**Background:**

Termites and their microbial gut symbionts are major recyclers of lignocellulosic biomass. This important symbiosis is obligate but relatively open and more complex in comparison to other well-known insect symbioses such as the strict vertical transmission of *Buchnera* in aphids. The relative roles of vertical inheritance and environmental factors such as diet in shaping the termite gut microbiome are not well understood.

**Results:**

The gut microbiomes of 66 specimens representing seven higher and nine lower termite genera collected in Australia and North America were profiled by small subunit (SSU) rRNA amplicon pyrosequencing. These represent the first reported culture-independent gut microbiome data for three higher termite genera: *Tenuirostritermes*, *Drepanotermes*, and *Gnathamitermes*; and two lower termite genera: *Marginitermes* and *Porotermes*. Consistent with previous studies, bacteria comprise the largest fraction of termite gut symbionts, of which 11 phylotypes (6 *Treponema*, 1 *Desulfarculus*-like, 1 *Desulfovibrio*, 1 *Anaerovorax*-like, 1 *Sporobacter*-like, and 1 *Pirellula*-like) were widespread occurring in ≥50% of collected specimens. Archaea are generally considered to comprise only a minority of the termite gut microbiota (<3%); however, archaeal relative abundance was substantially higher and variable in a number of specimens including *Macrognathotermes*, *Coptotermes*, *Schedorhinotermes*, *Porotermes*, and *Mastotermes* (representing up to 54% of amplicon reads). A ciliate related to *Clevelandella* was detected in low abundance in *Gnathamitermes* indicating that protists were either reacquired after protists loss in higher termites or persisted in low numbers across this transition. Phylogenetic analyses of the bacterial communities indicate that vertical inheritance is the primary force shaping termite gut microbiota. The effect of diet is secondary and appears to influence the relative abundance, but not membership, of the gut communities.

**Conclusions:**

Vertical inheritance is the primary force shaping the termite gut microbiome indicating that species are successfully and faithfully passed from one generation to the next via trophallaxis or coprophagy. Changes in relative abundance can occur on shorter time scales and appear to be an adaptive mechanism for dietary fluctuations.

**Electronic supplementary material:**

The online version of this article (doi:10.1186/s40168-015-0067-8) contains supplementary material, which is available to authorized users.

## Background

Co-evolution of microbial species with eukaryotic hosts is well known for obligate endosymbionts such as *Buchnera* in aphids [1] and *Wolbachia* in nematodes [2]. The importance of vertical inheritance is less clear in more open symbioses such as the microbiota of gastrointestinal tracts in which environmental perturbations and lateral transfer of organisms between hosts may play a more prominent role. Using culture-independent small subunit (SSU) rRNA-based community profiling, Ley et al. [3,4] found that both host phylogeny and diet shape gut microbiomes in many mammalian species and Ochman et al. concluded that vertical inheritance of gut microbiota in primates is discernable over evolutionary time scales [5].

Termites provide an appealing model system to explore the relative importance of vertical inheritance and environmental factors on symbiotic gut microbiota as unlike most insects, their gut communities are relatively complex comprising in the order of hundreds of species [6]. Termites are thought to have evolved from a cockroach-like ancestor into strictly eusocial insects that feed exclusively on lignocellulosic biomass [7]. Such recalcitrant substrates are digested through an obligate symbiosis with specialized gut microbiota comprising bacteria and protists in lower termites (classified into eight families) and bacteria only, in more recently evolved higher termites (classified in a single family, the Termitidae) [8]. Accordingly, transmission of gut microorganisms between termites is more strictly regulated than in mammals via trophallaxis (mouth to mouth transmission) or coprophagy (consumption of feces) [9] and co-speciation with the host has been observed in selected members of the gut community [9]. To determine whether vertical inheritance is the dominant force shaping termite gut communities more broadly, we used SSU rRNA gene amplicon pyrosequencing to profile the gut microbiomes of 66 termite samples, representing 16 genera, obtained in Australia and North America. These data expand current knowledge of termite gut microbiome diversity and represent the first gut community profiles for three higher (*Tenuirostritermes*, *Drepanotermes*, *Gnathamitermes*) and two lower (*Marginitermes*, *Porotermes*) termite genera.

## Results

### Sample collection and host identification

Samples of 66 termite colonies and two cockroaches were collected in Australia (Queensland and the Northern Territory) and the United States (Arizona) (Additional file 1: Table S1). Termites were identified by sequencing and comparative analysis of their mitochondrial cytochrome oxidase II (COII) genes [10] using the cockroaches as outgroup taxa. They were classified according to their closest identified phylogenetic neighbor in the public reference database (Additional file 2: Figure S1) and also by soldier morphology (Additional file 3: Figure S2). A total of 16 termite genera were sampled, seven higher and nine lower termites representing five of the nine recognized families (Table 1). The phylogenetic tree used to classify our samples (Additional file 2: Figure S1) is consistent with previous inferences based on COII and other marker genes [11-13] with the following observations. The genus *Nasutitermes* is not monophyletic [14], clustering together with several other nasute genera (subfamily Nasutitermitinae) including *Tumulitermes*, *Hospitalitermes*, and specimens 7TT2 and 7TT3, morphologically identified as *Tenuirostritermes*. Similarly, *Amitermes* is not monophyletic, clustering together with *Gnathamitermes* and *Drepanotermes*, although it should be noted that internal groupings within the Termitidae are not well supported by bootstrap resampling. Specimens 8MH1 and 9MH1 are the first COII data for the genus *Marginitermes*, and these sequences are grouped with members of the family Kalotermitidae as predicted by morphological similarities [15]. All other COII sequences obtained from the collected specimens, including cockroach outgroups, are grouped with reference sequences belonging to the expected genera predicted by morphology (Additional file 2: Figure S1). We then used this host phylogeny as a reference to establish the degree of vertical inheritance occurring with resident gut microbiomes.

**Table 1:** **Summary of the surveyed 66 termite whole gut samples according to host phylogeny (genus and family); sample location (country); and relative bacterial, archaeal, and protist abundances using universal primers (926F) and prokaryote primers (803F) in some instances (see text and Additional file** **13** **: Figure S10)**

**Termite genus**		**Family**	**Number of samples**			**Bacteria**		**Archaea**		**Protist**
			**Aus**	**USA**	**Total**	**%**		**%**		**%**
*Higher*						*926F*	*803F*	*926F*	*803F*	
1	*Drepanotermes*	Termitidae	1	0	1	97.5		2.5		0.0
2	*Gnathamitermes*	Termitidae	0	8	8	99.7		0.1		0.2
3	*Amitermes*	Termitidae	2	8	10	98.2		1.7		0.0
4	*Nasutitermes*	Termitidae	7	1	8	97.5		2.5		0.0
5	*Tenuirostritermes*	Termitidae	0	2	2	99.8		0.2		0.0
6	*Microcerotermes*	Termitidae	12	0	12	99.1		0.9		0.0
7	*Macrognathotermes*	Termitidae	1	0	1	77.9	63.5	22.1	36.5	0.0
		*Sub-total*	*24*	*18*	*42*					
*Lower*						*926F*	*803F*	*926F*	*803F*	
8	*Reticulitermes*	Rhinotermitidae	0	3	3	92.0		0.2		7.8
9	*Heterotermes*	Rhinotermitidae	6	0	6	91.4		5.3		3.3
10	*Coptotermes*	Rhinotermitidae	3	0	3	66.5	78.6	33.4	21.4	0.1
11	*Schedorhinotermes*	Rhinotermitidae	3	0	3	82.3	72.9	17.2	27.1	0.5
12	*Marginitermes*	Kalotermitidae	0	2	2	98.5		0.0		0.2
13	*Incisitermes*	Kalotermitidae	0	1	1	97.6		0.0		1.5
14	*Glyptotermes*	Kalotermitidae	2	0	2	100.0		0.0		2.4
15	*Porotermes*	Stolotermitidae	1	0	1	42.1	50.1	57.7	49.9	0.0
16	*Mastotermes*	Mastotermitidae	3	0	3	82.2	92.4	17.4	7.6	0.4
		*Sub-total*	*18*	*6*	*24*					
		*Overall*	*42*	*24*	*66*					

### Gut microbiome profiling

Whole guts were removed and pooled from 5 to 30 workers depending on the size of the species (Additional file 1: Table S1). In the case of the two cockroach outgroups, the gut material of a single individual was used for subsequent analyses. Culture-independent microbial community profiles were determined via SSU rRNA gene amplicon pyrosequencing using the primers 926F and 1392R that broadly target all three domains of life [16]. To evaluate the reproducibility of the profiles based on sets of pooled workers, we initially generated three biological replicates for four samples representing different termite genera. Clustering of samples by redundancy analysis (RDA) using Hellinger transformed data showed that the variation between the biological replicates of each subsampled genus was significantly less than the variation between termite genera (Additional file 4: Figure S3). Based on these observations and to permit a broader survey, we generated only one pooled worker sample profile for each of the remaining 62 termite specimens. A total of 457,947 pyrosequence raw reads were produced from the 68 samples ranging from 600 to 10,000 per sample after removal of termite (or cockroach) host SSU rRNA gene sequences, which comprised from 3% to 55% of total reads for each sample. Specimens were randomly resampled to a depth of 600 reads, and rarefaction and diversity analysis suggested that this was adequate to describe the overall diversity of the samples (Additional file 5: Figure S4). The resampled data was normalized for SSU rRNA copy number variation using CopyRighter [17] which can vary by up to an order of magnitude between prokaryotic genera. However, the effect of copy number correction was relatively subtle for these datasets (Additional file 6: Table S2). Overall, the majority of non-host amplicon reads from the whole gut samples were bacterial (95.4% on average in higher termites, 83.8% in lower) with smaller percentages of archaea (4.5% in higher, 14.4% in lower) and protists (0.1% in higher, 1.1% in lower) recovered (Table 1).

### Bacterial profiles

To determine the evolutionary distribution and conservation of bacterial groups across the sampled termite host radiation, we performed a prevalence versus relative abundance analysis [18]. Beginning at the broad taxonomic rank of phylum, all termite gut microbiomes were noted to comprise a core set (100% prevalence) of four bacterial phyla (Bacteroidetes, Firmicutes, Spirochaetes, and Proteobacteria) and an accessory set (<100% prevalence) of six bacterial phyla (Elusimicrobia, Fibrobacteres, Actinobacteria, Synergistetes, Planctomycetes, and Acidobacteria) using a relative abundance threshold of 1% in at least one sample (Table 2). Within the termite cohort, the core and accessory phyla showed pronounced differences in prevalence and relative abundances most notably between lower and higher termites. On average across the sampled genera, the Bacteroidetes are more abundant in lower than in higher termites, and the Spirochaetes, Acidobacteria, Fibrobacteres, and Synergistetes are more abundant in higher than lower termites (Table 2 and Additional file 7: Figure S5). We also observed that the Elusimicrobia are highly abundant in many lower termites while being nearly absent in all higher termites (Additional file 8: Figure S6). These differences in relative abundance are mostly accounted for by a small number of genera in each of the phyla (see below). Additionally, we noted a secondary pattern associated with diet at the phylum level. Polyphagous termite genera (i.e. those comprising species with different diets) tended to show an increase in the relative abundance of Spirochaetes and Fibrobacteres and a decrease of Firmicutes on a wood relative to a grass diet (*Nasutitermes*) and on a grass relative to dung diet (*Gnathamitermes*) (Additional file 9: Figure S7).

**Table 2:** **Summary of core and accessory bacterial phyla in higher and lower termite gut communities present at >1% relative abundance in at least one sample**

	**Higher**		**Lower**		
**Phylum** ^**a**^	**Prevalence**	**Relative abundance**	**Prevalence**	**Relative abundance**	***p*** **values**
	**%**	**% (SD)**	**%**	**% (SD)**	
**Bacteroidetes**	100.0	6.3 (±5.0)	100.0	41.3 (±24.8)	***
**Firmicutes**	100.0	24.0 (±14.1)	100.0	19.1 (±11.6)	_
**Spirochaetes**	100.0	44.3 (±18.9)	100.0	13.2(±13.0)	***
**Proteobacteria**	100.0	5.5 (±2.7)	100.0	7.5 (±6.5)	_
Planctomycetes	100.0	4.3 (±4.4)	79.1	2.3 (±2.6)	_
Synergistetes	95.2	3.1 (±3.0)	95.8	1.0 (±0.6)	_
Actinobacteria	92.9	1.8 (±1.8)	87.5	2.3 (±2.1)	_
Acidobacteria	90.5	2.0 (±1.3)	45.8	<1 (±0.8)	***
Fibrobacteres	95.2	5.7 (±5.2)	12.5	<1 (±1.1)	***
Elusimicrobia	31.0	<1 (±0.2)	70.8	8.4 (±15.3)	_

Prevalence and average relative abundance (and standard deviation) of each phylum in each group is shown. Statistically significant differences between phyla in higher and lower termites are indicated in the final column (see also Additional file 7: Figure S5).

*SD* standard deviation.

***p value <0.05.

_*p* value >0.05.

^a^Core phyla are bolded.

**Figure 1:**
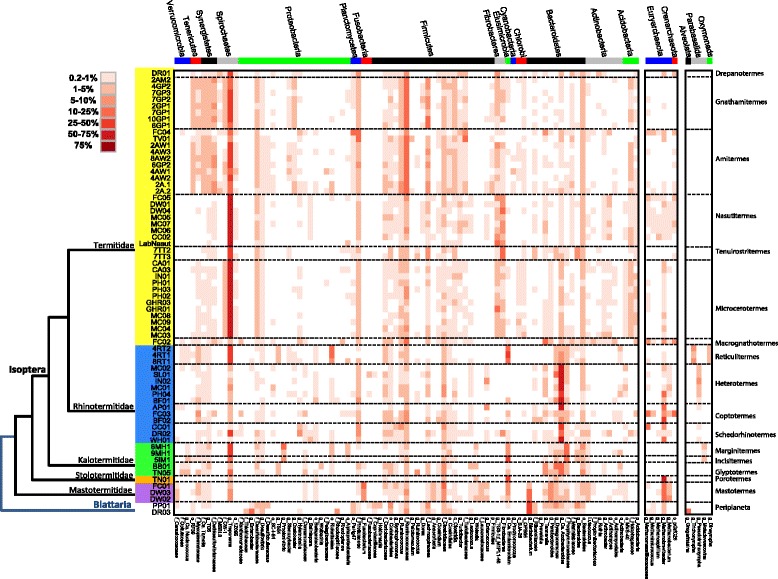
**Heatmap showing microbial taxa (mostly genus and family level) with relative abundance ≥0.2% in one or more whole gut samples surveyed in this study.** Each row represents a gut sample and each column a microbial taxon with relative abundance indicated by shading according to the legend. Phylum-level designations for the microbial taxa are indicated at the top of the figure, and host sample phylogeny is indicated to the left (family) and right (genus) of the figure.

**Figure 2:**
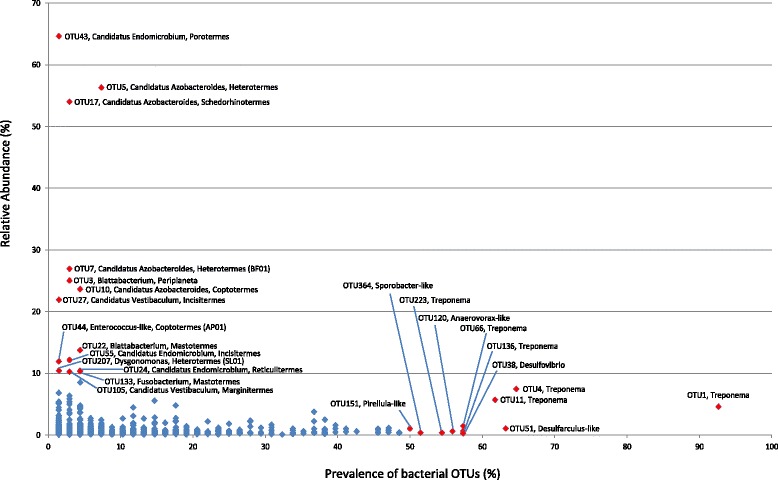
**Prevalence versus relative abundance graph of bacterial OTUs (97% sequence identity) in the surveyed gut samples.** OTUs with ≥10% relative abundance or ≥50% prevalence across the 66 termite samples are highlighted in red and labeled with OTU ID and closest matching bacterial genus. Relative abundance was calculated only using samples containing detectable amounts of a given OTU. In instances where the OTU is only found in a single termite genus, the termite genus is also included in the label.

For the Bacteroidetes, the genus *Candidatus* Azobacteroides is highly represented in many of the lower termite specimens; and for the Elusimicrobia, members of the genus *Candidatus* Endomicrobium are similarly highly represented in several lower termite genera (Figure 1). For the Spirochaetes, the genus *Treponema* is highly represented in all of the higher termite genera; and for the Fibrobacteres, which were not detected in most of the lower termite samples, members of the classes Chitinovibrionae (TG-3) and Fibrobacteres-2 were broadly represented in higher termite specimens (Figure 1). At increased phylogenetic resolution, several operational taxonomic units (OTUs) stood out either because they were abundant (>10% of bacterial reads) in one or a few termite genera and/or prevalent in the surveyed termites (present in >50% of specimens) (Figure 2). Four OTUs belonging to *Candidatus* Azobacteroides represent on average >10% of the reads from the guts of a number of lower termite genera and appear to have a co-evolutionary signal. For example, OTU5 is found in five of the six *Heterotermes* specimens that cluster together in the COII tree (Additional file 2: Figure S1), with the phylogenetic outlier, *Heterotermes* BF01 containing a different *Candidatus* Azobacteroides OTU (OTU7; Figure 2). Similarly, three abundant *Candidatus* Endomicrobium OTUs likely representing separate species occur in different lower termite genera (*Porotermes*—OTU43, *Incisitermes*—OTU55, *Reticulitermes*—OTU24; Figure 2 and Additional file 10: Figure S8). Other abundant OTUs included *Candidatus* Vestibaculum in *Incisitermes* (OTU27) and *Marginitermes* (OTU105), *Blattabacterium* in *Mastotermes* (OTU22) and in the cockroach outgroups (OTU3), *Enterococcus* (OTU44) in one *Coptotermes* sample (AP01), *Dysgonomonas* (OTU207) in one *Heterotermes* sample (SL01), and *Fusobacterium* (OTU133) in all three *Mastotermes* specimens. In terms of prevalence, *Treponema* was the standout genus, with six *Treponema* OTUs being broadly represented across the higher termites and in some instances also across the lower termites, for example OTU1 (present in 92% of all specimens; Figure 2). To confirm that the ubiquity of this OTU was not due to sample contamination, we examined it at higher resolution by dividing the 7,223 reads comprising OTU1 into identical clusters (Additional file 11: Table S3). Most (89%) of these identical clusters were from members of the same termite families suggesting minimal contamination (and vertical inheritance) and also indicating that while 97% OTUs reduce the effect of pyrosequencing error on diversity estimates [19], they are often composites of multiple strains [20]. Although OTU1 was present as a low abundance member in most termite genera (<1%), it was highly represented in *Microcerotermes* (up to 35% of bacterial reads; Additional file 9: Figure S7). Other high prevalence (and mostly low abundance) OTUs included *Desulfarculus*-like (OTU51), *Desulfovibrio* (OTU38), *Anaerovorax*-like (OTU120), *Sporobacter*-like (OTU364), and *Pirellula*-like (OTU151) bacteria (Figure 2 and Additional file 10: Figure S8).

### Archaeal profiles

**Figure 3:**
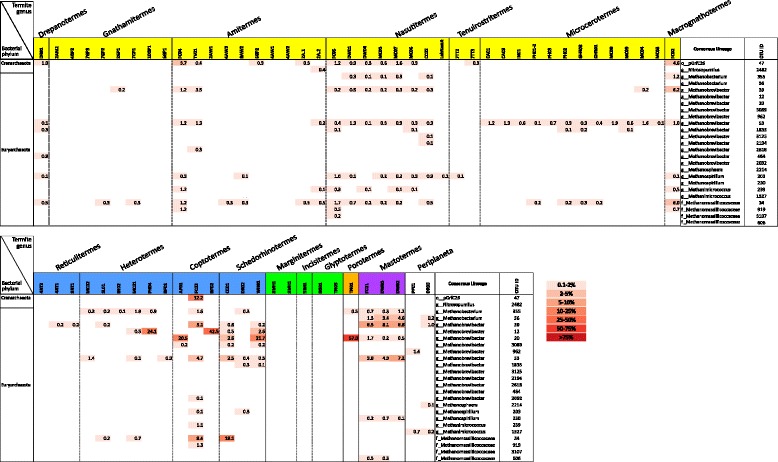
**Heatmap showing archaeal OTUs (97% seq id) with ≥0.1% relative abundance in one or more of the surveyed gut samples.** Each row represents an OTU and each column a gut sample with relative abundance as a percentage of the total microbial community (including bacteria) indicated by numbers and shading according to the legend. The termite genus for each sample is indicated at the top of the figure, and OTU phylogeny is indicated to the left (phylum) and right (mostly genus) of the figure.

Archaea comprise a minority of the higher termite gut community profiles with the exception of the *Macrognathotermes* sample (20% of reads) and represent >10% of the profiles in four of the nine lower termite genera investigated, in one instance comprising more than half the reads (*Porotermes* 57%; Table 1). Three of the five termite genera with high archaeal signal had multiple representatives (*Coptotermes, Schedorhinotermes,* and *Mastotermes*), which showed a high degree of variation in the percentage of archaeal reads (Figure 3). To cross-check that this variation and that the unexpectedly high archaeal abundance in many of these samples was not the result of primer bias, we generated additional community profiles using a different forward primer, 803F, which broadly targets bacteria and archaea [16]. The profiles were largely consistent between the two primer sets confirming both the sample-to-sample variation within a termite genus and that the archaea comprise a high percentage of the amplicon reads in some samples (Table 1 and Additional file 12: Figure S9). The majority of detected archaeal phylotypes are Euryarchaeota most closely related to methanogenic genera including (in descending relative abundance) *Methanobrevibacter, Methanomassiliicoccus, Methanobacterium, Methanimicrococcus,* and *Methanospirillum.* Additionally, a Crenarchaeote belonging to an uncultured lineage, pGrfC26 [21], was detected up to 10.2% in some termite genera (Figures 1 and 3).

### Eukaryotic profiles

Non-termite host eukaryotic sequences represented only 1.0% of the community profiles averaged over the 16 termite genera with the highest fraction recovered in the lower termite *Reticulitermes* (8%; Table 1). These percentages likely do not reflect protist cell numbers or ratios due to the much higher number of rRNA copies in protists relative to bacteria and variation of copy number between protist lineages [22]. The majority of the eukaryotic reads were classified as parabasalids (*Trichonympha, Pseudotrichonympha*, and *Metadevescovina*) and oxymonads (*Dinenympha*) (Figure 1 and Additional file 13: Figure S10). A low abundance phylotype (0.2 to 0.5%) most closely related to the ciliate *Clevelandella* (98% sequence identity) was unexpectedly detected in half of the *Gnathamitermes* samples (Additional file 13: Figure S10).

### Beta-diversity analyses

**Figure 4:**
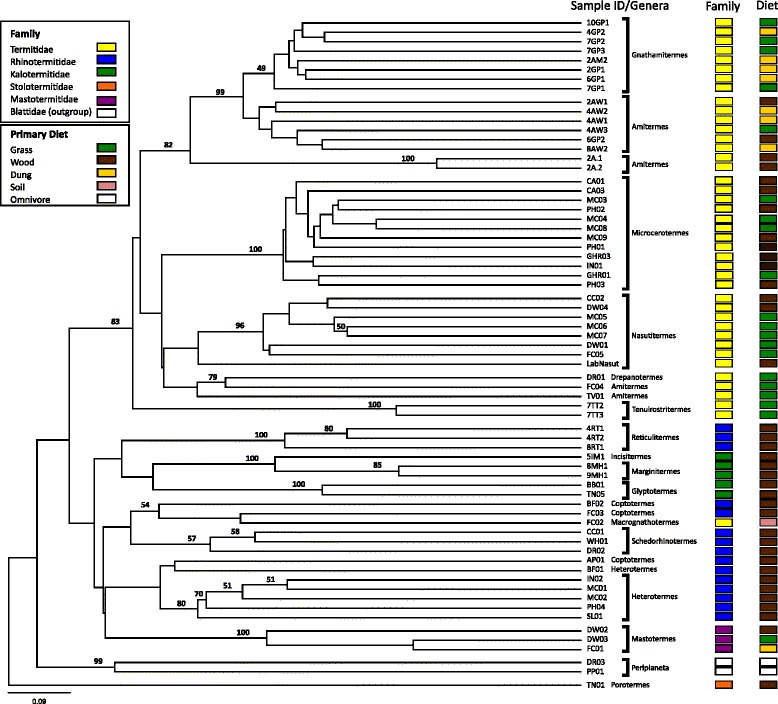
**UPGMA tree of unweighted (presence/absence only) Soergel pairwise distances between bacterial profiles showing a high consistency with host phylogeny and low consistency with diet (Additional file** **14** **: Table S4).** The values on interior nodes represent jackknife support values ≥49. Termite host affiliation (family) and presumptive diet are indicated to the right of the tree.

**Figure 5:**
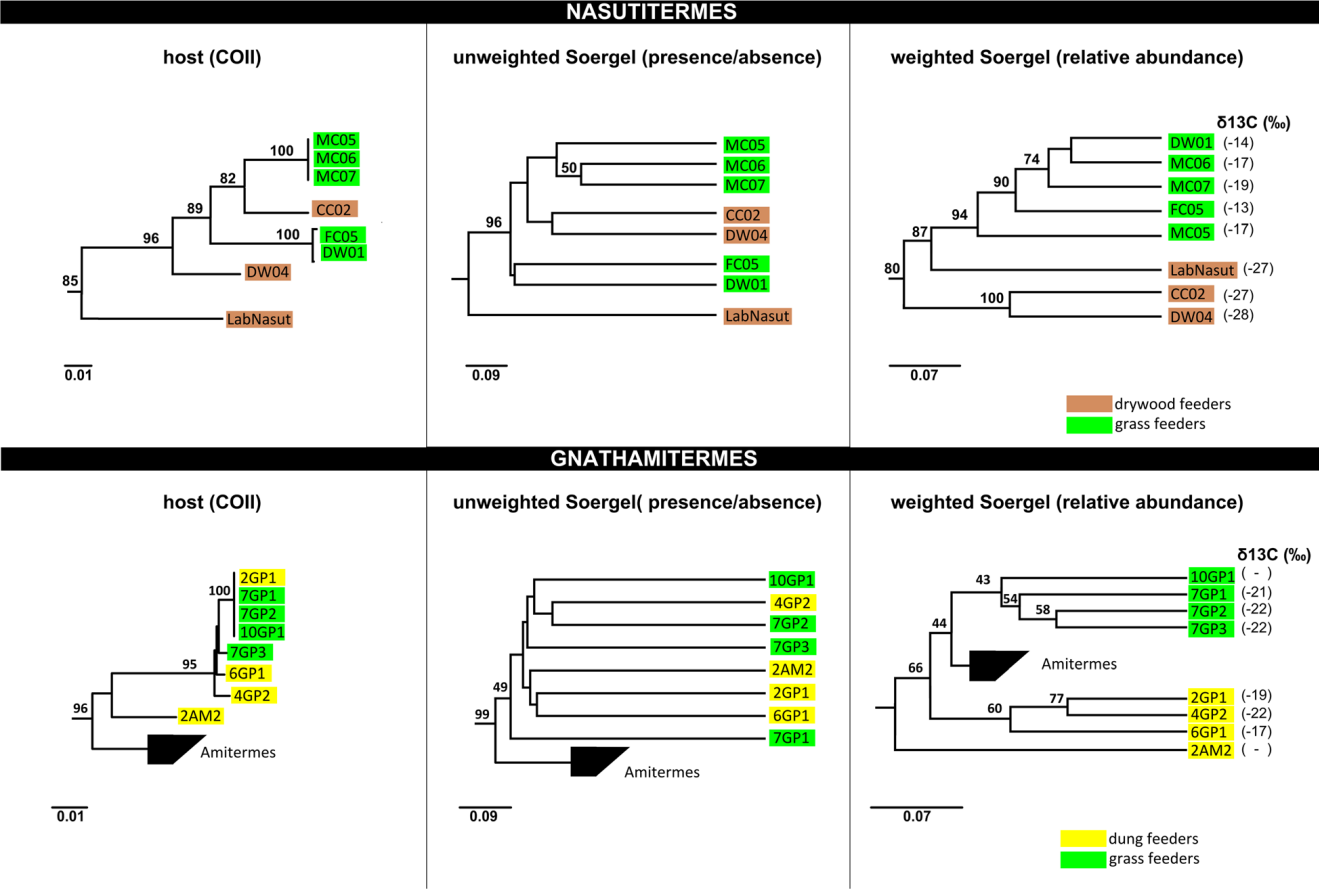
**Subtrees of host and bacterial community phylogenetic comparisons showing secondary effect of diet on community structure of polyphagous termite genera.** When the relative abundance of bacterial OTUs is taken into account (weighted Soergel), samples cluster according to diet. The values on interior nodes of the COII trees are FastTree local support values and jackknife support values ≥49 on the Soergel UPGMA Carbon isotope values of gut contents are shown in the far right panels.

To explore the relative effect of vertical inheritance and diet on termite gut microbiota, we calculated phylogenetic distances between bacterial communities with (weighted) or without (unweighted) taking OTU relative abundance into account. Hierarchical clustering of unweighted Soergel dissimilarity distances produced a topology highly consistent with the inferred host evolution (Additional file 2: Figure S1; [11]) but not with inferred diet where dietary variation was present, that is, in polyphagous genera (Figure 4 and Additional file 14: Table S4). All termite genera with >1 representative were resolved as monophyletic groups according to comparison of their gut bacteria with the exception of *Coptotermes* and *Amitermes* (Figure 4)*.* However, the latter genus was also not monophyletic within the COII tree (Additional file 2: Figure S1), with FC04 and TV01 forming a separate line of descent in both trees. The vertical inheritance signal was strong enough to resolve some family level associations (with >1 genus), including the Termitidae with the exception of *Macrognathotermes* and the Kalotermitidae (Figure 4). When OTU relative abundance was taken into account, the host signal was weakened particularly at the family level, but most termite genera were still resolved as monophyletic groups (Additional file 15: Figure S11). Closer inspection of *Nasutitermes* and *Gnathamitermes* revealed that relative abundance clustered members of these polyphagous genera by diet (Figure 5) reflecting the phylum-level shifts noted previously (Additional file 9: Figure S7). Isotopic analysis of gut contents supports this observation as putative wood feeders had isotopically heavier carbon (d13C:−25‰ to −27‰) than their grass (d13C: −13‰ to −14‰) or dung feeding (d13C: −16‰ to −22‰) counterparts (Figure 5).

## Discussion

Termite gut microbiota have been the subject of an increasing number of investigations over the past years using a suite of new molecular tools [7]; however, a large amount of termite diversity remains to be explored. Here, we present the first extensive culture-independent molecular survey of the gut microbiomes of Australian termites and expand our existing knowledge of North American termite gut microbial diversity. These data are then used to assess the relative effect of vertical inheritance and environmental factors (primarily diet). The 16 termite genera examined in this study have a set of core and accessory gut bacterial phyla that distinguish them from all other habitats (Table 2). This observation is consistent with previous culture-independent studies which show that the combination of these phyla is highly distinctive of the termite gut microbiome [7,23-25] particularly in comparison to other insect gut communities [26,27]. This distinctiveness is further underlined by the observation that the majority of operational taxonomic units (OTUs) identified in the present study cluster with sequences from previous termite surveys [28-38]. A recent extensive rRNA-based survey of gut bacteria in 34 termite species [24] allows direct comparison of the bacterial profiles of seven termite genera that overlap between the studies. The three higher termite profiles generally match well, but the four lower termite profiles have some conspicuous differences even at the relatively course phylogenetic resolution of phylum. In particular, the Dietrich et al. [24] profiles have higher proportions of Spirochaetes and lower proportions of Bacteroidetes and Firmicutes than the corresponding profiles in our study (Additional file 16: Figure S12). For *Reticulitermes* and *Coptotermes*, this may reflect real differences as different species were profiled; but for *Mastotermes* and *Incisitermes* for which the same species were examined, the more likely explanation is differences arising from methodology such as DNA extraction method [39] and/or PCR primers used [40]. A study by Sabree and Moran [41] using similar DNA extraction method and primers to ours produced a similar gut community profile for *Mastotermes* (Additional file 16: Figure S12).

With these methodological caveats in mind, key differences between higher and lower termite gut profiles are linked to the presence of protists in the latter group. The Bacteroidetes and Elusimicrobia are the most over-represented phyla in lower termites relative to higher termites because they harbor highly abundant members of the *Candidatus* genera Azobacteroides*,* Vestibaculum (Bacteroidetes), and Endomicrobium (Elusimicrobia; Figure 2), which are recognized protist symbionts [9,42,43]. *Candidatus* Azobacteroides pseudotrichonymphae, an endosymbiont of the parabasalid *Pseudotrichonympha grasii,* has previously been reported to comprise approximately 70% of the bacterial cells present in the gut of *Coptotermes formosanus* [44]. Here, we found phylogenetically distinct *Candidatus* Azobacteroides spp. comprise up to 66% of the bacterial gut profiles in *Coptotermes,* up to 63% in *Schedorhinotermes*, and up to 72% in *Heterotermes* (Additional file 10: Figure S8) and identified their putative *Pseudotrichonympha* hosts only in those termite genera (Figure 1 and Additional file 13: Figure S10), supporting the previously reported specific relationship between the two in multiple termite genera [9]. *Candidatus* Vestibaculum illigatum was first reported in *Neotermes cubanus* and was shown to be an epibiont of the flagellated protist *Staurojoenina* [45]. Here, we found abundant populations (8%–22%) of *Candidatus* Vestibaculum in *Incisitermes* and *Marginitermes*, both members of the family Kalotermitidae. Assuming that *Candidatus* Vestibaculum is a specific epibiont of *Staurojoenina*, this is consistent with the observation that *Staurojoenina* is only found in members of the family Kalotermitidae [46,47]. However, the other Kalotermitidae genus surveyed, *Glyptotermes*, lacked detectable populations of *Candidatus* Vestibaculum (Additional file 10: Figure S8) and *Staurojoenina* was not detected at all in our survey. The latter observation may be due to our primer set not targeting this parabasalid genus (two mismatches in the 926F primer to *S. assimilis* acc. AB183882).

*Candidatus* Endomicrobium was detected in all of the lower termite genera surveyed and was also found in low abundance in some of the higher termite genera (Figure 1) consistent with previous findings [48-50]. In *Reticulitermes* and *Incisitermes*, *Candidatus* Endomicrobium is a recognized cytoplasmic symbiont of the parabasalids *Trichonympha* and *Metadevescovina*, respectively [50]. Our data are consistent with these observations as high abundance populations of *Candidatus* Endomicrobium, and their respective host protists were detected in *Reticulitermes* and *Incisitermes* (Figure 1). The highest relative abundance of *Candidatus* Endomicrobium was found in *Porotermes* (65% of bacterial reads; Figure 2 and Additional file 10: Figure S8); however, no protist host sequences were detected, presumably due to primer mismatches as visual observation of *Porotermes* gut contents reveal a high diversity of protist morphotypes (unpublished observation).

The most prevalent (ubiquitous) genus in the gut survey was *Treponema* (Figure 2), which comprises most of the Spirochaetes phylum signal*. Treponema* has been reported in every termite gut investigation to date [7] and contributes substantially to the distinctiveness of the termite gut microbiome. Numerous *Treponema* OTUs were found in the present survey, many of which flourished in the higher termites (Additional file 10: Figure S8) likely following the evolutionary loss of protists from the hindgut [51]. It has been shown that Spirochaetes are essential for the survival of higher termites and that their removal results in a shorter life span [52]. Metagenomic, metatranscriptomic, and metaproteomic analyses of two higher termite genera, *Nasutitermes* and *Amitermes,* indicate that *Treponemas* are involved in all of the major functions in the hindgut, including fiber hydrolysis, fermentation, homoacetogenesis, and nitrogen fixation [23,53] which may explain their success (ubiquity) and long term co-habitation with their termite hosts. However, *Treponema* is a phylogenetically broad genus [54-56] and it seems likely that not all species will be capable of all key functions.

Two Deltaproteobacteria (*Desulfovibrio* and *Desulfarculus*-like OTUs) were present in low abundance in over half of the samples tested (Figure 2). *Desulfovibrio* has previously been reported as a widespread constituent of termite guts mainly based on cultivation studies, with proposed functions including oxygen removal and nitrogen fixation [57]. However, the *Desulfarculus*-like OTU was more prevalent (Additional file 10: Figure S8) and a member of this group has recently been inferred to be primarily responsible for the first step in CO_2_-reductive acetogenesis [58]. If this key functionality in the *Desulfarculus*-like group is conserved across different termite genera, it may explain their widespread distribution among the surveyed termites. Less expected was the widespread occurrence of a *Pirellula*-like planctomycete OTU (Figure 2). Planctomycetes have been reported in alkaline gut segments of soil-feeding termite genera, where they are speculated to play a role in degradation of humus-associated biopolymers such as N-acetylglucosamine [32]. No soil-feeding genera were surveyed in the present study, although the planctomycete OTU may be associated with alkaline segments known to be present in several higher termite genera [59]. The planctomycete OTU was also detected in three lower termite genera (Additional file 10: Figure S8) which are not known to have alkaline gut segments, suggesting that planctomycetes are not strictly associated with higher pH in termites.

Archaea have been reported to constitute only a small fraction (up to 3%) of the termite gut ecosystem [60]; however, we found much higher percentages in the amplicon profiles of a number of lower termite genera and one higher termite genus (Table 1). We cross-checked our findings with an alternative forward primer broadly targeting bacteria and archaea (803F) and confirmed that the result was not an artifact of the universal primer pair (926F and 1392R). Also considering that many samples had archaeal proportions in the anticipated range (<3%; Table 1), we suggest that the higher values are not artifacts of the primers or of the DNA extraction method used. The observed variability in archaeal abundance between samples belonging to the same termite genus, e.g. *Schedorhinotermes* (1.4%, 24.5%, and 32.9%), suggests that archaeal abundance may be more variable between specimens than previously appreciated, possibly reflecting environmental factors or simply temporal dynamics (‘archaeal blooms’). Only hydrogenotrophic methanogens, dominated by *Methanobrevibacter* in most cases, were detected in the surveyed termite guts consistent with previous reports [61-63], suggesting that acetoclastic methanogenesis is likely unfavorable in this habitat. Phylotypes closely related to a recently described phylogenetically novel methanogenic genus related to the Thermoplasmatales, *Methanomassiliicoccus*, were detected in several termite genera raising the possibility that these methanogenic populations may have an obligate requirement for methanol [64].

Eukaryotes were not the primary focus of this study, and our data are likely an underestimate of protist diversity in the surveyed species due to primer mismatches [65,66]. Also, rRNA-based relative abundance estimates will likely not reflect cell counts (e.g. *Reticulitermes* [67]) due to the much higher number of rRNA gene copies in protists relative to bacteria [22], the former of which is not currently corrected by CopyRighter [17]. However, some interesting qualitative observations were made including putative protist host-bacterial symbiont pairings described above. It is commonly reported that higher termites lack flagellated protists, which are primary agents of lignocellulose digestion in lower termites [7,68,69]. Instead, bacteria and to a lesser extent, the termite itself, provide the enzymes necessary for lignocellulose hydrolysis in higher termites [7,70]. Unexpectedly then, a phylotype related to the ciliate *Clevelandella,* previously reported in wood-feeding cockroach intestinal tracts [71], was detected in the higher termite genus *Gnathamitermes* (Additional file 13: Figure S10)*.* An older microscopic study of higher termite gut ecosystems supports our findings with the identification of small numbers of a closely related ciliate, *Nyctotherus*, in *Amitermes* [72], although no protists were detected in the *Amitermes* community profiles in the present study. The presence of low abundance protist populations in some higher termite genera suggests either reacquisition after the major evolutionary transition to bacteria-dominated gut communities in the higher termites or low-level persistence of some protist species across this transition. It will be interesting to determine if these ciliates are directly involved in lignocellulose digestion.

A primary motivation of our study was to determine the relative importance of vertical inheritance (host signal) versus diet on termite gut microbiota composition given the unusual status of termites among insects in terms of gut microbiome complexity [6] and the importance of termites as ecosystem engineers [23]. This question is not immediately addressable using field observations of lower termites as they are primarily wood feeders with the exception of *Mastotermes* [73]. However, we obtained sufficient specimens of polyphagous higher termite genera to evaluate the relative effect of diet and host signal. The strongest signal was clearly due to vertical inheritance, with termite genus and even family level associations being resolved based on gut community profiles alone, particularly in unweighted analyses (Figure 4). This is consistent with previous studies indicating that vertical transmission plays an important role in structuring termite gut communities, for example co-speciation of gut symbionts within the genera *Reticulitermes* and *Microcerotermes* [28] and a general host signal in whole gut community analyses of 34 termite and cockroach species [24]. Maintenance of host-specific microbial communities must be achieved via vertical transmission during trophallaxis or coprophagy, as there is no germline transfer in termites [74]. It is important to note that a dominant host signal in gut community composition does not imply that all component species are the product of vertical inheritance, ultimately resulting in co-speciation. The termite gut is an open system that would allow ingress of foreign microorganisms, which may be able to persist under favorable conditions. For example, it was speculated that some Firmicute populations in *Amitermes* have been laterally acquired from herbivore gut communities as a result of dietary specialization, i.e. dung feeding [23] (see below). These bacterial populations were then subsequently vertically transmitted in the *Amitermes* lineage. While fine-scale reconstruction of population co-evolution is not feasible with partial rRNA sequences, the clusters of identical reads identified in the most ubiquitous 97% OTU, *Treponema* OTU1, reflects the dominant overall host signal but also suggests that a minority of strains in the cluster may have been laterally transferred between termite genera (Additional file 11: Table S3).

The effect of diet on gut community structure has been addressed to a lesser extent in termites. No clear dietary signal was observed in unweighted analyses (Figure 4), but when the evenness (relative abundance) of gut phylotypes was taken into account, a secondary effect of diet on community structure became apparent in the well-sampled polyphagous termite genera. Specifically, *Nasutitermes* samples are partitioned into wood- and grass-feeding clades and *Gnathamitermes* into grass- and dung-feeding clades (Figure 5). Changes in phylum-level abundances could be correlated with the dietary differences such as an increased abundance of Spirochaetes and Fibrobacteres and decreased abundance of Firmicutes in wood-feeding relative to grass-feeding *Nasutitermes* (Additional file 9: Figure S7). This is consistent with previous reports of the importance of Spirochaetes and Fibrobacteres in the digestion of wood fibers [53,75]. He et al. [23] identified phylum-level shifts between dung-feeding *Amitermes* and wood-feeding *Nasutitermes.* Based on metagenomic and metatranscriptomic analyses, they explained these differences by inferring that Firmicutes play a greater role in hemicellulose hydrolysis and utilization of fixed-nitrogen compounds required for dung digestion and Spirochaetes play a greater role in cellulose hydrolysis and nitrogen fixation required for wood digestion. However, our data suggest that phylum-level differences attributed to diet were overestimated in the He et al. study [23] because of marked differences between the *Amitermes* and *Nasutitermes* gut communities due to vertical inheritance. We estimate that changes in the relative abundance of these phyla between wood- and grass-feeding *Nasutitermes* samples is only 4%–8%, as opposed to the 15%–34% differences seen between dung-feeding *Amitermes* and wood-feeding *Nasutitermes* (Additional file 9: Figure S7). Presumably in some instances, changes in relative abundances of gut populations occurred over evolutionary time scales in response to dedicated dietary specialization [76]. However, recent feeding trial studies of *Reticulitermes flavipes* indicate that such changes in population evenness can occur on short time scales allowing polyphagous termite species to adjust rapidly to changes in their diet due to seasonal variation or availability of foraged plant species [77,78].

## Conclusions

In summary, we infer that vertical inheritance is the primary force shaping the termite gut microbiome and that most indigenous species are successfully and faithfully passed from one termite generation to the next. Changes in relative abundance can occur on shorter time scales and appear to be an adaptive mechanism for changes in diet. The resilience of termite gut communities to experimental dietary perturbations remains to be fully explored. Our findings suggest that an evolutionary perspective will greatly assist in deconvoluting specific and whole community functionality in the termite gut microbiome.

## Methods

### Sample collection and processing

Termite collections were made on public lands in Queensland, Northern Territory (Australia), and Arizona (United States of America). Where possible, specimens were collected with their nest material and transported to the laboratory in ventilated plastic containers at room temperature to reduce stress to the insects. Termites were removed from their nest material within a day of arriving in the laboratory. For community profiling, workers were transferred to a metal tray and frozen at −80°C for 20 min, then collected into 2 ml cryotubes and stored at −20°C until further processing. Frozen specimens were thawed on ice, and gut tracts were extracted using clean sharp tweezers. The guts were immediately transferred into a sterile 1.5 ml microtube on ice and stored at −20°C until extraction. For morphological identification, soldier specimens were stored in 85% ethanol.

### DNA extraction

Total genomic DNAs were extracted from pooled (5–30) whole gut samples, depending on size of species, using FastDNA® SPIN kit for Soil (MP Biomedicals, Australia). Termite guts were added to a lysing matrix, treated with lysis buffer, and underwent bead beating in the Vortex-Genie® 2 (MoBio Laboratories, USA). DNA was bound to silica matrix and washed and eluted in DNase-free water. DNA yield was then quantified by the Qubit™ fluorometer and QuantIT ds-DNA BR assay kit (Invitrogen, Australia). DNA concentration varied depending on the biomass of the whole gut. DNA concentrations were standardized across all samples to 20 μg/ml, diluting where necessary in Ultrapure™ distilled water (Invitrogen, Australia). DNA quality was evaluated using gel electrophoresis on 1.0% agarose gels stained with SYBR Safe, visualized on a CCD compact image system (Major Science, USA).

### SSU rRNA PCR and amplicon pyrosequencing

The universal primer pair 926F (or prokaryote-specific 803F) and 1392R were used to amplify the V6 to V8 variable regions of the SSU rRNA gene. Primer sequences were modified by incorporation of the Roche 454 A or B adaptor sequences and a unique 5–7 nucleotide barcode (multiplex identifier; MID) to identify amplicons originating from different samples in the same sequencing reaction. The reverse primer 1392R was barcoded on the 5′end with the *MID* between the 454 A adaptor (uppercase) and the SSU rRNA primer (lowercase) (5′-CCA TCT CAT CCC TGC GTG TCT CCG AC TCAG [MID] acgggcggtgtgtRc-3′); and the 926 forward primer (lowercase) was modified by addition of 454 B adaptor (uppercase) at its 5′end (3′-CCT ATC CCC TGT GTG CCT TGG CAG TC TCAG aaactYaaaKgaattgRcgg-3′) (or 803 forward primer ttagaKacccBNgtagtc) [40].

DNA amplification was carried out in 50 μl PCR reactions, using 2 μl of termite whole gut DNA extract as template. The amplification mixture contained 0.2 μl of 1U Fisher BioReagents* *Taq* DNA polymerase (Thermo Fisher Scientific Inc., USA), 4 μl of 25 mM MgCl_2_, 1.5 μl of BSA (Roche diagnostic, Australia), 5 μl of 10X buffer, 1 μl of dNTP mix (each at a concentration of 10 mM), 1 μl of each 10 mM forward primer and reverse primer; and *E.coli* was used as the positive control. PCR was performed using a Veriti® Thermal Cycler (Applied Biosystems™, Australia) with the following cycling parameters: an initial denaturation step of 95°C for 3 min followed by 30 cycles of 95°C for 30 s, 55°C for 45 s, 72°C for 90 s, and a final extension of 72°C for 10 min. Amplification products were quantified by electrophoresis in 1% agarose with SYBR Safe staining.

To ensure that similar numbers of sequencing reads were obtained for each sample, PCR amplicons were pooled in equal concentrations after cycling and then purified using the Agencourt® AMPure® XP Kit (Beckman, USA). DNA was quantified with the Qubit™ fluorometer and QuantIT ds-DNA BR assay kit. Cleaned, pooled, barcoded amplicons were submitted for pyrosequencing library preparation where they were mixed in equal proportions with other samples prior to emulsion PCR for GS FLX pyrosequencing (454 Life Sciences, USA).

### Analysis of SSU rRNA gene sequences

SSU rRNA sequence data were obtained from the multiplexed 454 run by converting the pyrosequencing flowgrams to sequence reads using the standard software provided by 454 Life Sciences [19,40]. Short and/or low quality reads were removed using UCHIME version 4.2 [79], and homopolymer errors were corrected using Acacia [80]. Sequence data were analyzed using a pyrotag (pyrosequence reads) processing pipeline, Quantitive Insights Into Microbial Ecology (QIIME) [81] and CD-HIT [82]. Reads were hard trimmed to 250 bp and clustered into operational taxonomic units (OTUs) with a threshold of 97% sequence identity using MCL [83]. OTU representatives were compared to the Greengenes database (February 2011 release) for taxonomy assignment using BLAST [84,85]. A table which lists the relative abundance of each OTU in each sample was generated and visualized as a heatmap. The relationship between the microbial communities in different samples was assessed using jackknifed UPGMA trees derived from the distance matrices obtained with the phylogeny-based unweighted and weighted Soergel beta-diversity measures implemented in Express Beta Diversity v1.04 [86]. The Soergel distance measures community relatedness based on phylogeny and either presence/absence (unweighted) or relative abundance (weighted) of OTUs [87-89]. A comparative analysis of several phylogenetic beta-diversity measures resulted in the recommendation of the Soergel measure based in part on the unweighted variant being identical to unweighted UniFrac and the weighted variant being closely related to normalized, weighted UniFrac [86]. The relative abundance of different phyla within the higher and lower termites was compared using Welch’s *t*-tests with Šidák multiple test correction as implemented in STAMP [90].

### Molecular identification of termite host species

The mitochondrial cytochrome oxidase II (COII) gene was amplified with PCR using three sets of primers Fleu/Rlys (TCT AAT ATG GCA GAT TAG TGC/GAG ACC AGT ACT TGC TTT CAG TCA TC), COIIF-M13/COIIR-M13 (GTT TTC CCA GTC ACG ACG TTG TAC AGA TAA GTG CAT TGG ATT T/AGG AAA CAG CTA TGA CCA TGG TTT AAG AGA CCA GTA CTT G), and COIIFw-M13/COIIRw-M13 (GTT TTC CCA GTC ACG ACG TTG TAC AGA YWA GTG CAH TGG ATT T/AGG AAA CAG CTA TGA CCA TGG TTT AAG AGA CCA KTA CTT G). This gene is commonly used for identification of termite species [11,91]. The amplification products were purified and directly sent for Sanger sequencing (Macrogen Inc., Korea). The sequences were manually trimmed and inspected using Geneious software (www.geneious.com). Reference COII nucleotide sequences were obtained from the National Center for Biotechnology Information server (http://www.ncbi.nlm.nih.gov) and aligned using Clustal W in ARB [92], followed by manual checking and refinement of the automated alignment. Nucleotide and amino acid-based trees were constructed using a neighbor-joining method in ARB, and the topologies were compared. The COII tree was inferred using FastTree v2.1.3 [93] with the generalized time-reversible model of nucleotide evolution.

### Nucleotide sequence accession numbers

All SSU rRNA sequence data obtained from this study have been deposited in GenBank under BioProject PRJNA248567. The COII termite host sequences are deposited under the accession numbers KJ907786–KJ907853.

## Additional files

Additional file 1: Table S1. Details of the 68 specimens collected in the present study. Additional file 2: Figure S1. Maximum likelihood (FastTree) tree of aligned mitochondrial cytochrome oxidase (COII) genes from termite samples included in this study (in blue) and publicly available reference sequences. Family level affiliations are indicated by color according to the legend at left. Additional file 3: Figure S2. Soldier morphologies of several termite specimens collected in Australia. Additional file 4: Figure S3. Redundancy analysis (RDA) plots of microbial profiles obtained from biological replicates of four termite genera. Differences were significantly less between biological replicates than between genera. Additional file 5: Figure S4. Rarefaction curves and associated Shannon diversity indices (H) of microbial profiles obtained for each of the 66 samples separated into different panels by termite genus affiliation. Additional file 6: Table S2. Effect of rRNA copy number correction on termite gut profile relative abundance estimates for OTUs with ≥1% in at least one sample. Additional file 7: Figure S5. Core and accessory bacterial phyla with a significant difference in mean proportions ≥1% between higher and lower termites and a *p* value ≤0.05. Statistical significance was assessed using Welch’s *t*-test with Šidák multiple test correction. Additional file 8: Figure S6. Relative proportion of Elusimicrobia across the higher and lower termite samples. Termite genus affiliations of the samples is shown to the left of the figure. Additional file 9: Figure S7. Average whole gut microbial community profiles of the 16 termite genera surveyed in this study. The profiles of the polyphagous termite genera *Gnathamitermes* and *Nasutitermes* are further divided by diet (in colored boxes). Additional file 10: Figure S8. Heatmap of bacterial OTUs (97% seq id) with ≥10% relative abundance or ≥50% prevalence across the 66 termite samples (Figure 2). Each row represents an OTU and each column a gut sample with relative abundance as a percentage of the total microbial community indicated by numbers and shading according to the legend. The termite genus and family for each sample is indicated at the top and bottom of the figure, respectively, and OTU phylogeny is indicated to the left (phylum) and right (mostly genus) of the figure. Additional file 11: Table S3. Distribution of identical sequence clusters comprising >10 reads in *Treponema* OTU1 (comprising 7,223 reads in total). Each row represents an identical cluster and each column a gut sample with absolute numbers of reads for each cluster and sample shown. The termite genus and family for each sample and country of origin are indicated at the top of the table using color coding. Additional file 12: Figure S9. Heatmap of archaeal OTUs generated with two primer pairs in whole gut samples of termites with ≥10% archaeal relative abundance (Table 2). Each row represents a different OTU, and the abundance as a percentage of the total community is indicated by shading according to the legend. Termite family affiliations of each sample are indicated at the top the figure, respectively, and OTU phylogeny is indicated to the left (phylum) and right (mostly genus) of the figure. Additional file 13: Figure S10. Heatmap of protist OTUs (97% seq id) across the 66 termite samples. Each row represents an OTU and each column a gut sample with relative abundance as a percentage of the total microbial community indicated by numbers and shading according to the legend. The termite genus and family for each sample is indicated at the top and bottom of the figure, respectively, and OTU phylogeny is indicated to the left (phylum) and right (mostly genus) of the figure. Additional file 14: Table S4. Consistency analysis of microbial community relationships based on weighted and unweighted Soergel distances with host phylogeny (COII) and presumptive diet. Additional file 15: Figure S11. UPGMA tree of weighted (relative abundance taken into account) Soergel pairwise distances between bacterial profiles showing a drop in consistency with host phylogeny (particularly family level) relative to the unweighted analysis (Figure 4; Additional file 14: Table S4). The values on interior nodes represent jackknife support values ≥49. Termite host affiliation (family) is indicated to the right of the tree. Additional file 16: Figure S12. Comparison of termite gut bacterial profiles obtained in the present study (rRNA copy number corrected and uncorrected profiles) and by [24]. An additional *Mastotermes* profile reported by [41] is also included for reference. For each study, the profiles are averaged across samples belonging to the same termite genus (number of samples is shown above each bar).

## Abbreviations

DNA: deoxyribonucleic acid
dNTPs: deoxynucleoside triphosphate
MID: multiplex identifiers
OTU: operational taxonomic unit
PCR: polymerase chain reaction
SSU rRNA: small subunit ribosomal RNA
