# Supplementary material for: A molecular survey of Australian and North American termite genera indicates that vertical inheritance is the primary force shaping termite gut microbiomes
Source: Microbiome. 2015 Feb 25;3:5. doi: 10.1186/s40168-015-0067-8 (PMC4379614; doi:10.1186/s40168-015-0067-8)
Supplement: Additional file 2: Figure S1. — Maximum likelihood (FastTree) tree of aligned mitochondrial cytochrome oxidase (COII) genes from termite samples included in this study (in blue) and publicly available reference sequences. Family level affiliations are indicated by color according to the legend at left. [file 40168_2015_67_MOESM2_ESM.pdf]

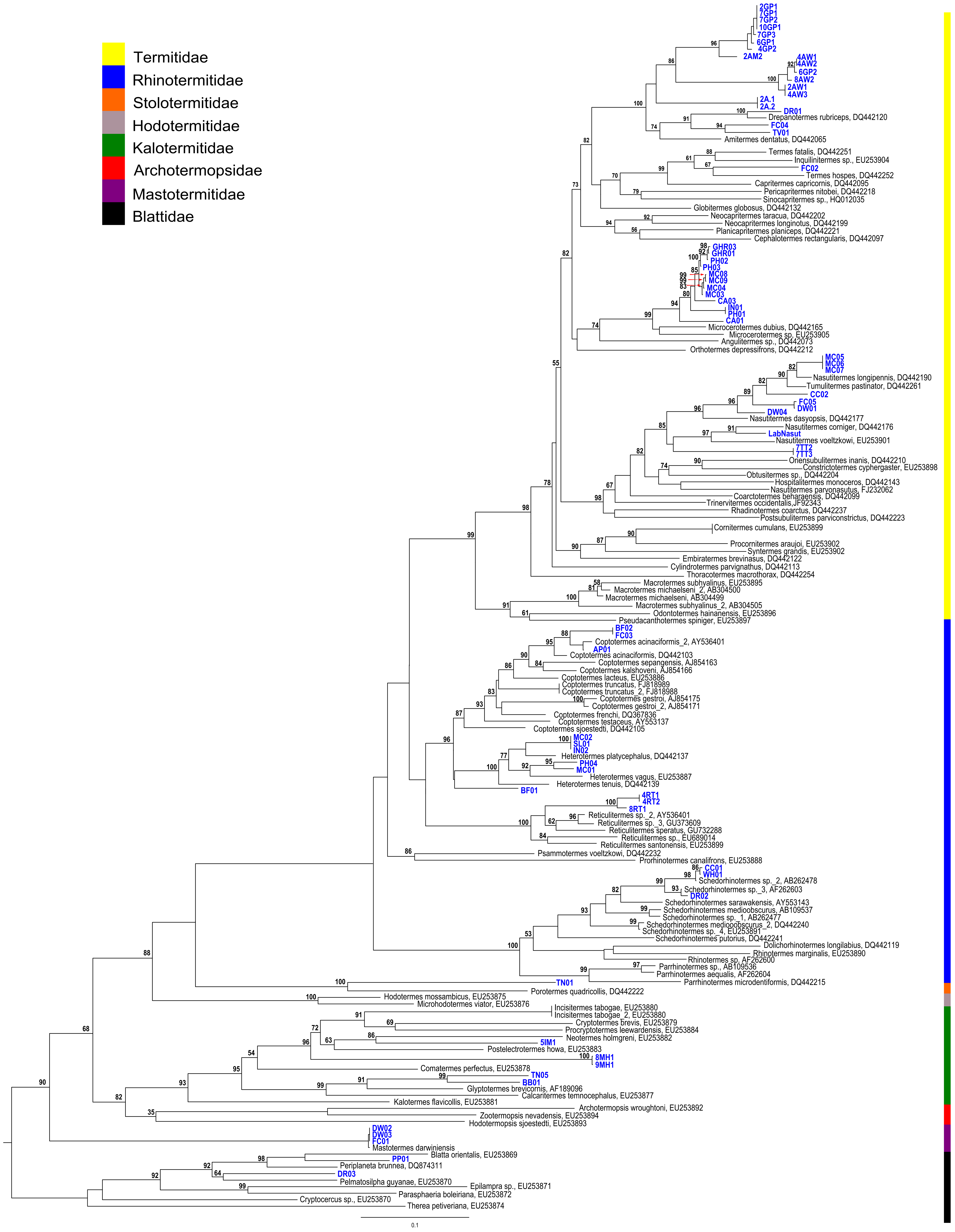
Termitidae
Rhinotermitidae
Stolotermitidae
Hodotermitidae
Kalotermitidae
Archotermopsidae
Mastotermitidae
Blattidae
2GP1
7GP1
7GP2
10GP1
7GP3
6GP1
4GP2
2AM2
4AW1
4AW2
6GP2
8AW2
2AW1
4AW3
2A.1
2A.2
DR01
Drepanotermes rubriceps, DQ442120
FC04
TV01
Amitermes dentatus, DQ442065
Termes fatalis, DQ442251
Inquilinitermes sp., EU253904
FC02
Termes hospes, DQ442252
Capritermes capricornis, DQ442095
Pericapritermes nitobei, DQ442218
Sinocapritermes sp., HQ012035
Globitermes globosus, DQ442132
Neocapritermes taracua, DQ442202
Neocapritermes longinotus, DQ442199
Planicapritermes planiceps, DQ442221
Cephalotermes rectangularis, DQ442097
GHR03
GHR01
PH02
PH03
MC08
MC09
MC04
MC03
CA03
IN01
PH01
CA01
Microcerotermes dubius, DQ442165
Microcerotermes sp, EU253905
Angulitermes sp., DQ442073
Orthotermes depressifrons, DQ442212
MC05
MC06
MC07
Nasutitermes longipennis, DQ442190
Tumulitermes pastinator, DQ442261
CC02
FC05
DW01
DW04
Nasutitermes dasyopsis, DQ442177
Nasutitermes corniger, DQ442176
LabNasut
Nasutitermes voeltzkowi, EU253901
7TT2
7TT3
Oriensubulitermes inanis, DQ442210
Constrictotermes cyphergaster, EU253898
Obtusitermes sp., DQ442204
Hospitalitermes monoceros, DQ442143
Nasutitermes parvonasutus, FJ232062
Coarctotermes beharaensis, DQ442099
Trinervitermes occidentalis,JF92343
Rhadinotermes coarctus, DQ442237
Postsubulitermes parviconstrictus, DQ442223
Cornitermes cumulans, EU253899
Procornitermes araujoi, EU253902
Syntermes grandis, EU253902
Embiratermes brevinasus, DQ442122
Cylindrotermes parvignathus, DQ442113
Thoracotermes macrothorax, DQ442254
Macrotermes subhyalinus, EU253895
Macrotermes michaelseni_2, AB304500
Macrotermes michaelseni, AB304499
Macrotermes subhyalinus_2, AB304505
Odontotermes hainanensis, EU253896
Pseudacanthotermes spiniger, EU253897
BF02
FC03
Coptotermes acinaciformis_2, AY536401
AP01
Coptotermes acinaciformis, DQ442103
Coptotermes sepangensis, AJ854163
Coptotermes kalshoveni, AJ854166
Coptotermes lacteus, EU253886
Coptotermes truncatus, FJ818989
Coptotermes truncatus_2, FJ818988
Coptotermes gestroi, AJ854175
Coptotermes gestroi_2, AJ854171
Coptotermes frenchi, DQ367836
Coptotermes testaceus, AY553137
Coptotermes sjoestedti, DQ442105
MC02
SL01
IN02
Heterotermes platycephalus, DQ442137
PH04
MC01
Heterotermes vagus, EU253887
Heterotermes tenuis, DQ442139
BF01
4RT1
4RT2
8RT1
Reticulitermes sp._2, AY536401
Reticulitermes sp._3, GU373609
Reticulitermes speratus, GU732288
Reticulitermes sp., EU689014
Reticulitermes santonensis, EU253899
Psammotermes voeltzkowi, DQ442232
Prorhinotermes canalifrons, EU253888
CC01
WH01
Schedorhinotermes sp._2, AB262478
Schedorhinotermes sp._3, AF262603
DR02
Schedorhinotermes sarawakensis, AY553143
Schedorhinotermes medioobscurus, AB109537
Schedorhinotermes sp._1, AB262477
Schedorhinotermes medioobscurus_2, DQ442240
Schedorhinotermes sp._4, EU253891
Schedorhinotermes putorius, DQ442241
Dolichorhinotermes longilabius, DQ442119
Rhinotermes marginalis, EU253890
Rhinotermes sp. AF262600
Parrhinotermes sp., AB109536
Parrhinotermes aequalis, AF262604
Parrhinotermes microdentiformis, DQ442215
TN01
Porotermes quadricollis, DQ442222
Hodotermes mossambicus, EU253875
Microhodotermes viator, EU253876
Incisitermes tabogae, EU253880
Incisitermes tabogae_2, EU253880
Cryptotermes brevis, EU253879
Procryptotermes leewardensis, EU253884
Neotermes holmgreni, EU253882
5IM1
Postelectrotermes howa, EU253883
8MH1
9MH1
Comatermes perfectus, EU253878
TN05
BB01
Glyptotermes brevicornis, AF189096
Calcaritermes temnocephalus, EU253877
Kalotermes flavicollis, EU253881
Archotermopsis wroughtoni, EU253892
Zootermopsis nevadensis, EU253894
Hodotermopsis sjoestedti, EU253893
DW02
DW03
FC01
Mastotermes darwiniensis
Blatta orientalis, EU253869
PP01
Periplaneta brunnea, DQ874311
DR03
Pelmatosilpha guyanae, EU253870
Epilampra sp., EU253871
Parasphaeria boleiriana, EU253872
Cryptocercus sp., EU253870
Therea petiveriana, EU253874
0.1
