# Supplementary figures and images for: A molecular survey of Australian and North American termite genera indicates that vertical inheritance is the primary force shaping termite gut microbiomes
Source: Microbiome. 2015 Feb 25;3:5. doi: 10.1186/s40168-015-0067-8 (PMC4379614; doi:10.1186/s40168-015-0067-8)

## Drepanotermes

DR01

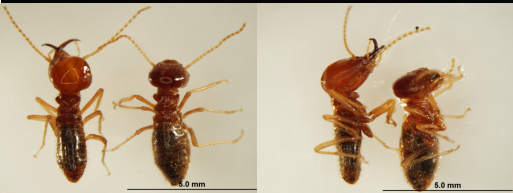

## Amitermes

FC04

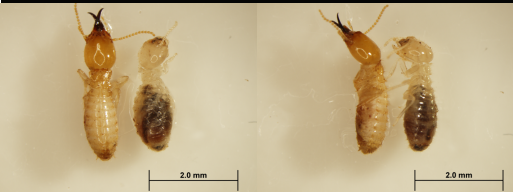

TV01

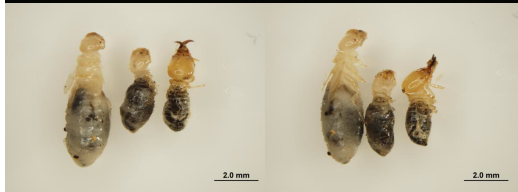

## Nasutitermes

FC05

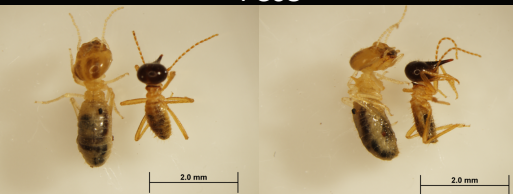

DW01

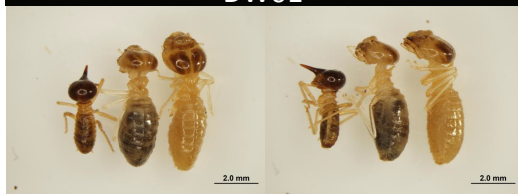

DW04

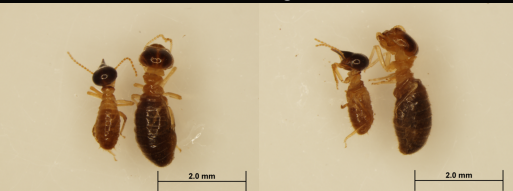

MC05

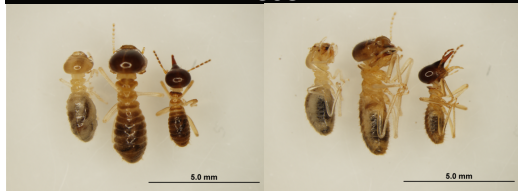

MC06

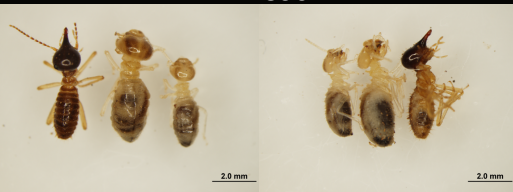

MC07

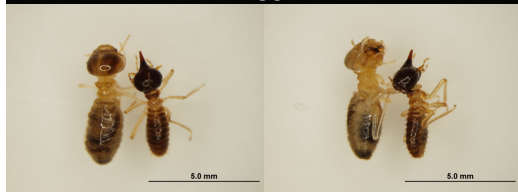

CC02

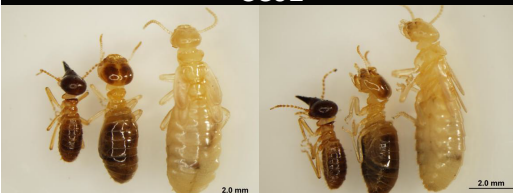

Supplement: Additional file 3: Figure S2. — Soldier morphologies of several termite specimens collected in Australia. [file 40168_2015_67_MOESM3_ESM.zip › 40168_2015_67_add3/40168_2015_67_add3a.pdf]

# Microcerotermes

CA01

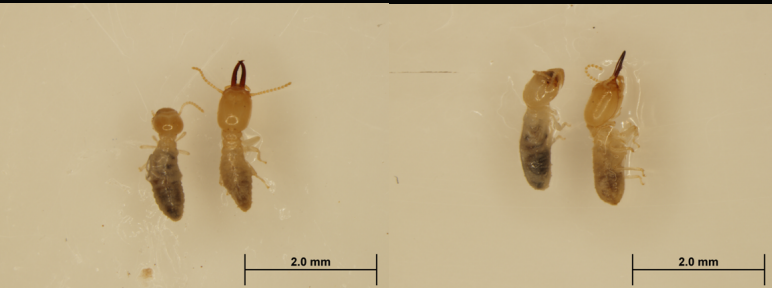

CA03

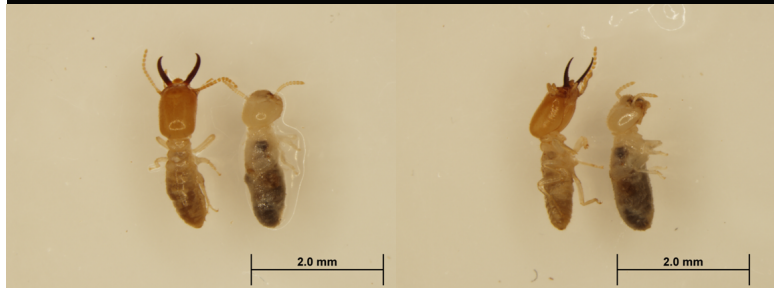

IN01

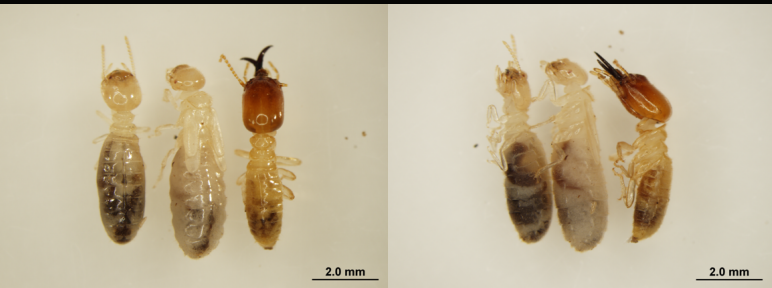

PH01

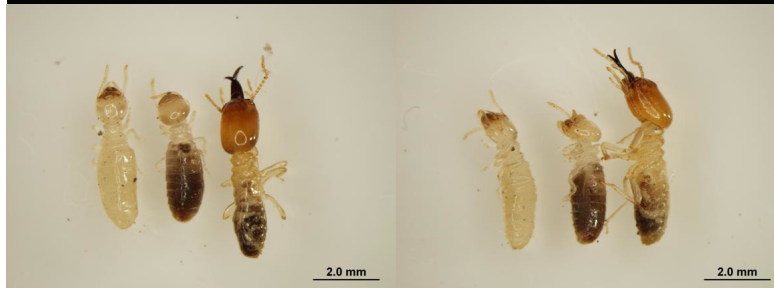

GHR01

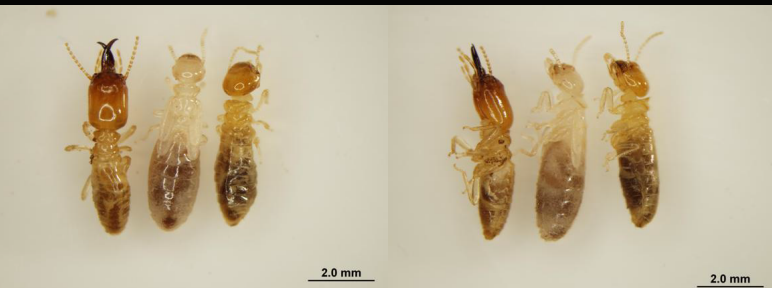

GHR03

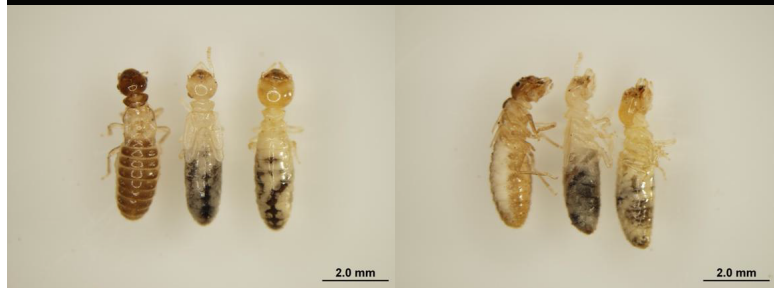

MC04

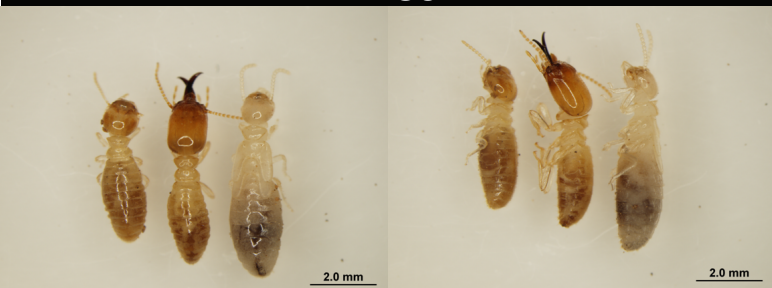

MC08

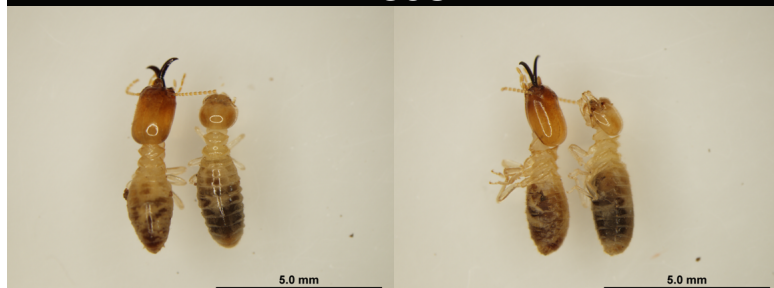

MC09

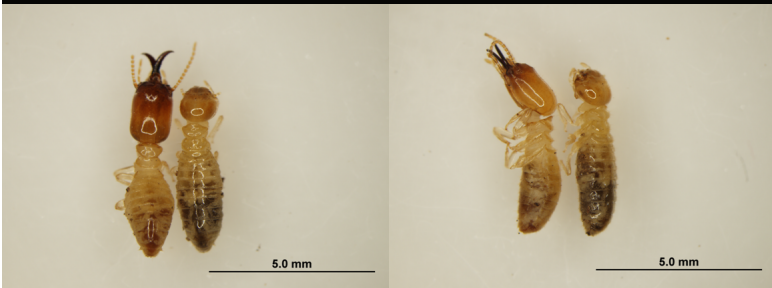

MC03

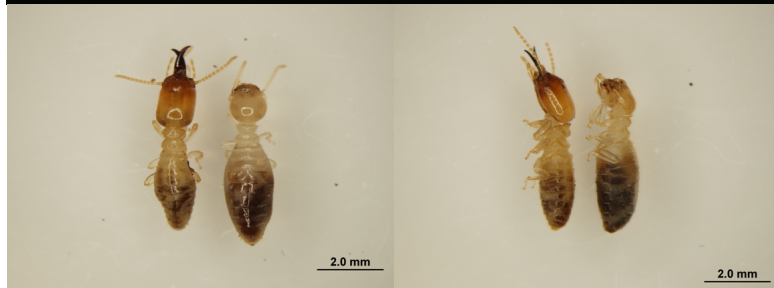

PH02

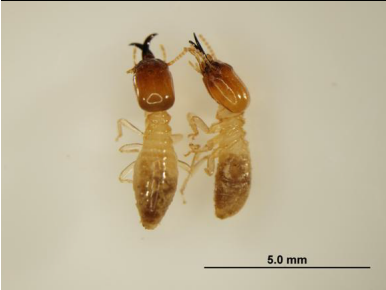

Supplement: Additional file 3: Figure S2. — Soldier morphologies of several termite specimens collected in Australia. [file 40168_2015_67_MOESM3_ESM.zip › 40168_2015_67_add3/40168_2015_67_add3b.pdf]

Macrognathotermes

FC02

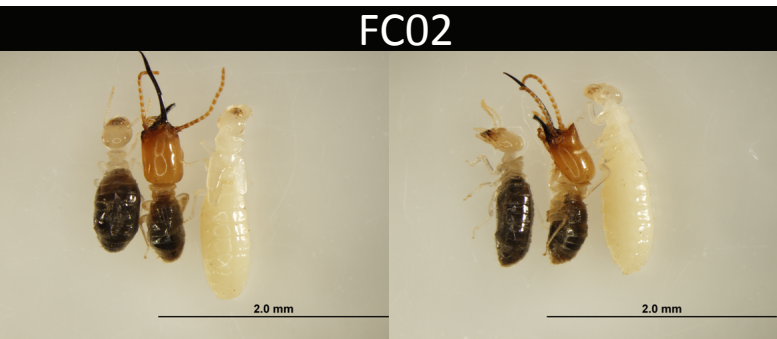

Heterotermes

MC02

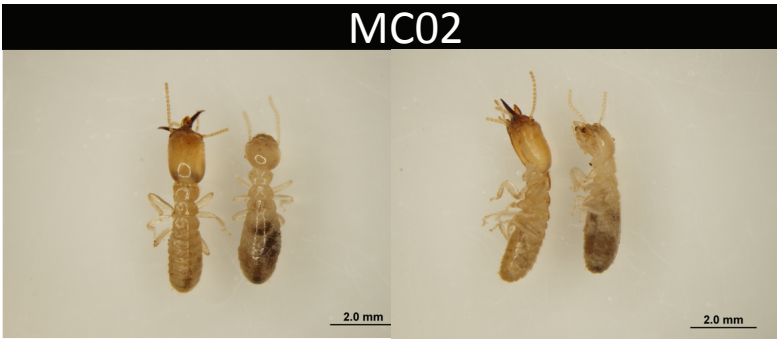

SL01

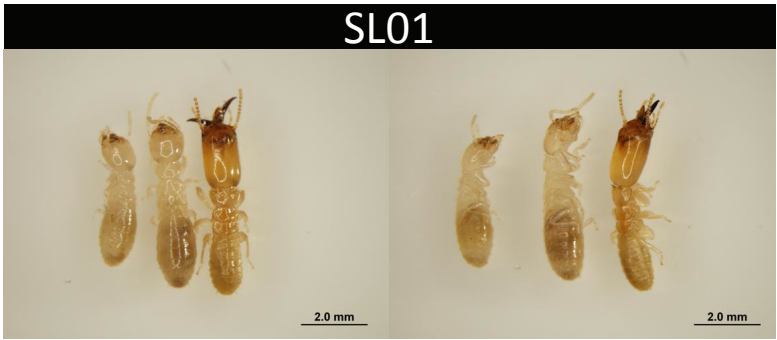

BF01

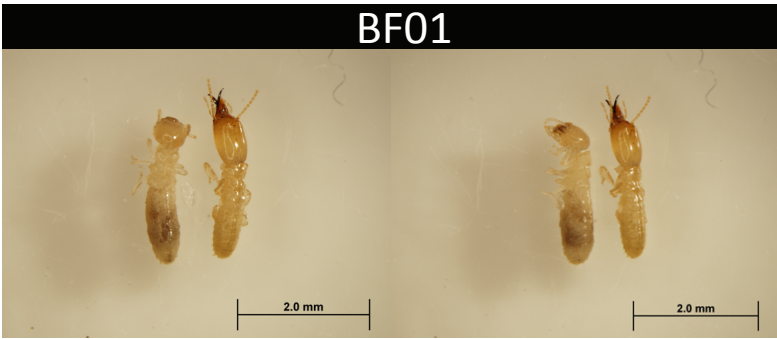

IN02

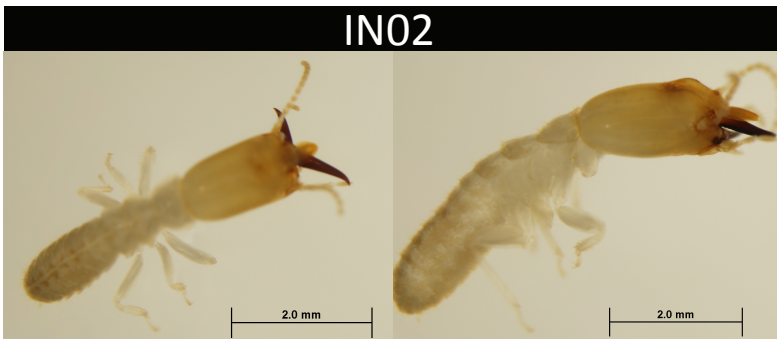

MC01

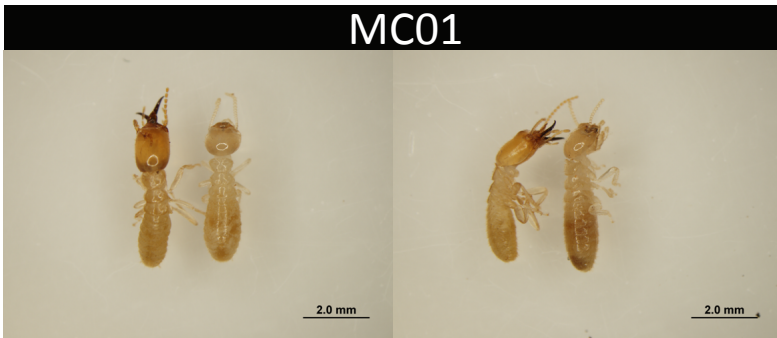

Coptotermes

BF02

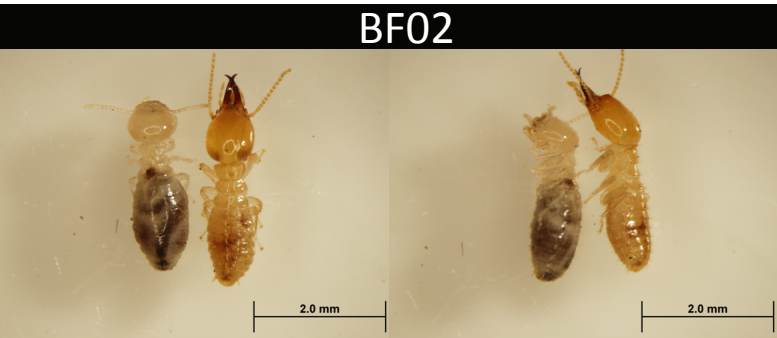

FC03

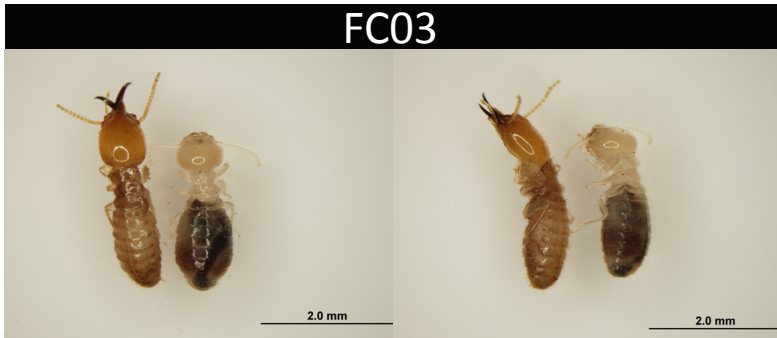

Schedorhinotermes

DR02

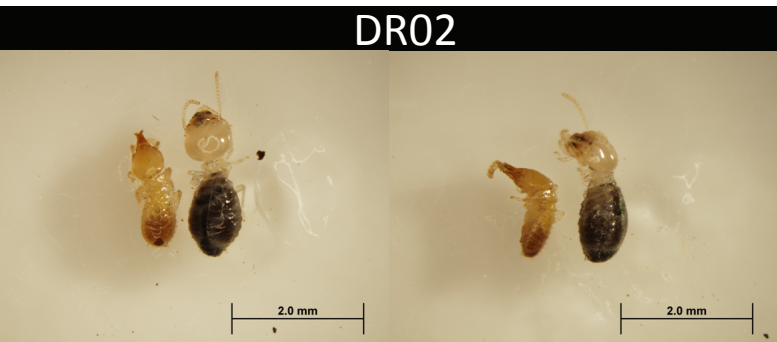

CC01

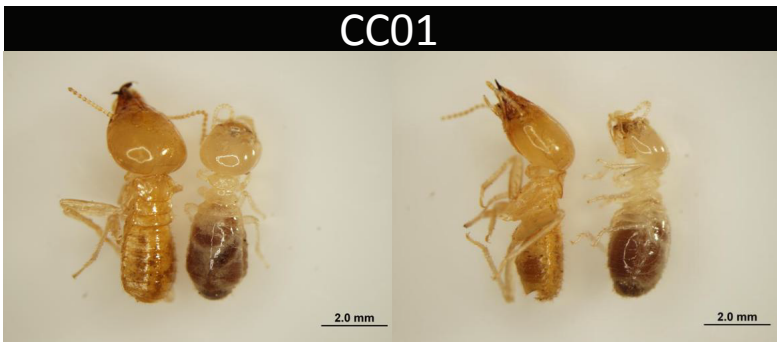

Supplement: Additional file 3: Figure S2. — Soldier morphologies of several termite specimens collected in Australia. [file 40168_2015_67_MOESM3_ESM.zip › 40168_2015_67_add3/40168_2015_67_add3c.pdf]

WH01

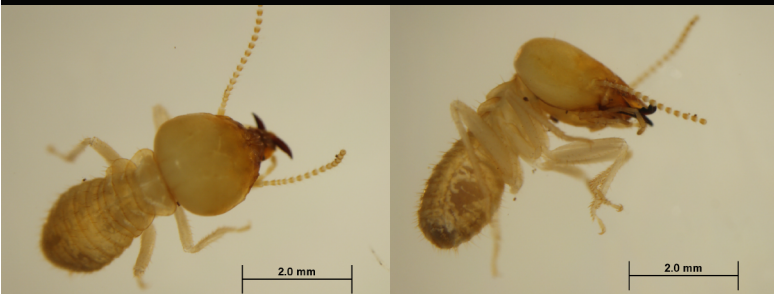

## Glyptotermes

BB01

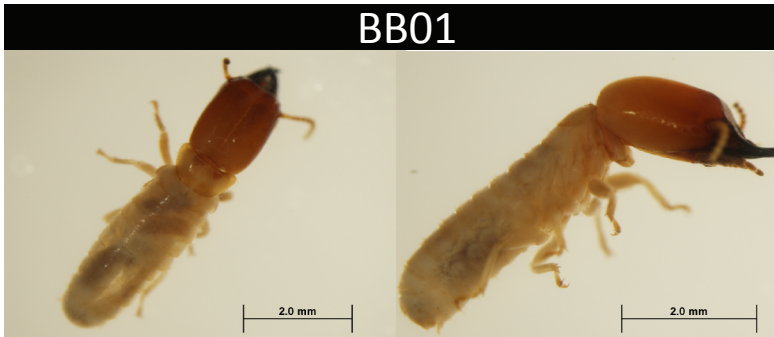

TN05

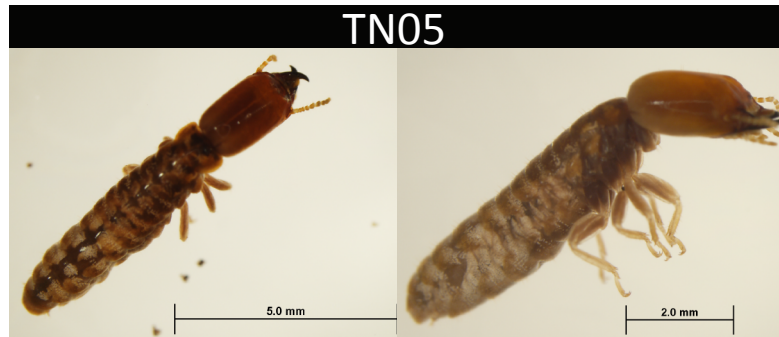

## Porotermes

TN01

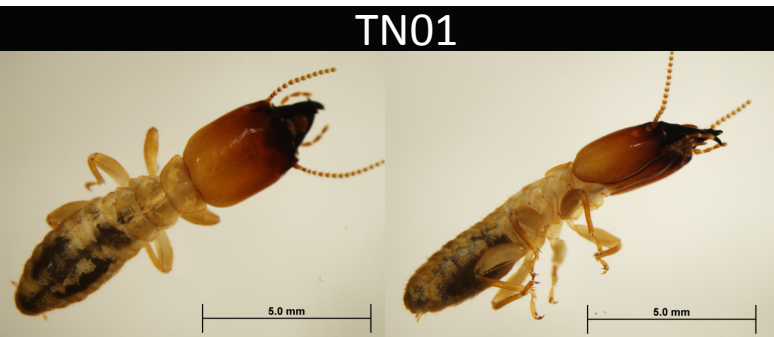

## Mastotermes

DW03

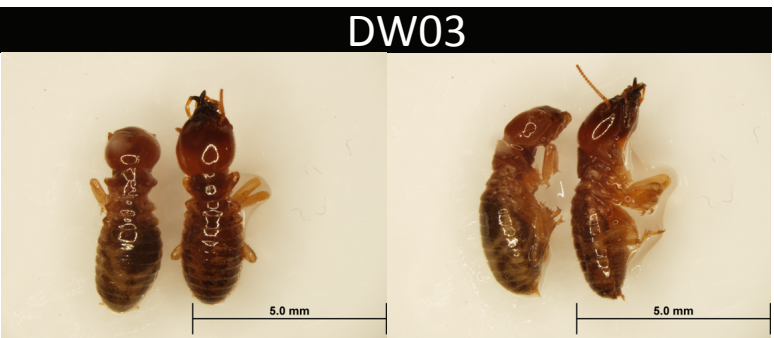

DW02

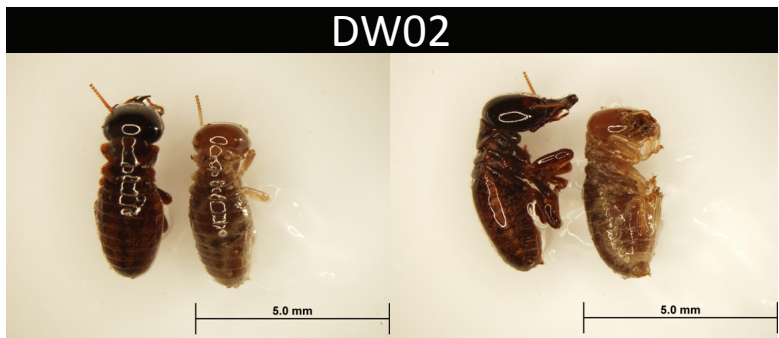

FC01

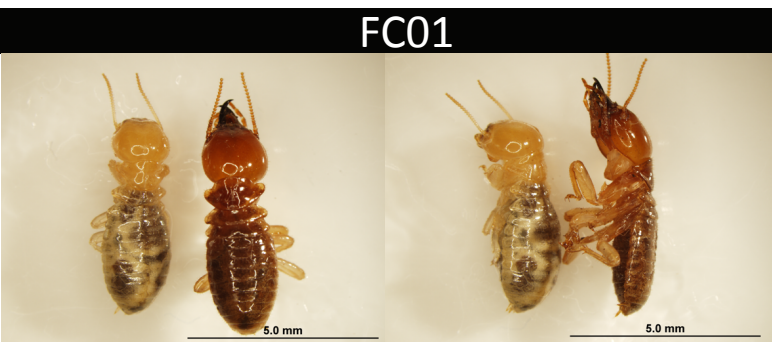

Supplement: Additional file 3: Figure S2. — Soldier morphologies of several termite specimens collected in Australia. [file 40168_2015_67_MOESM3_ESM.zip › 40168_2015_67_add3/40168_2015_67_add3d.pdf]

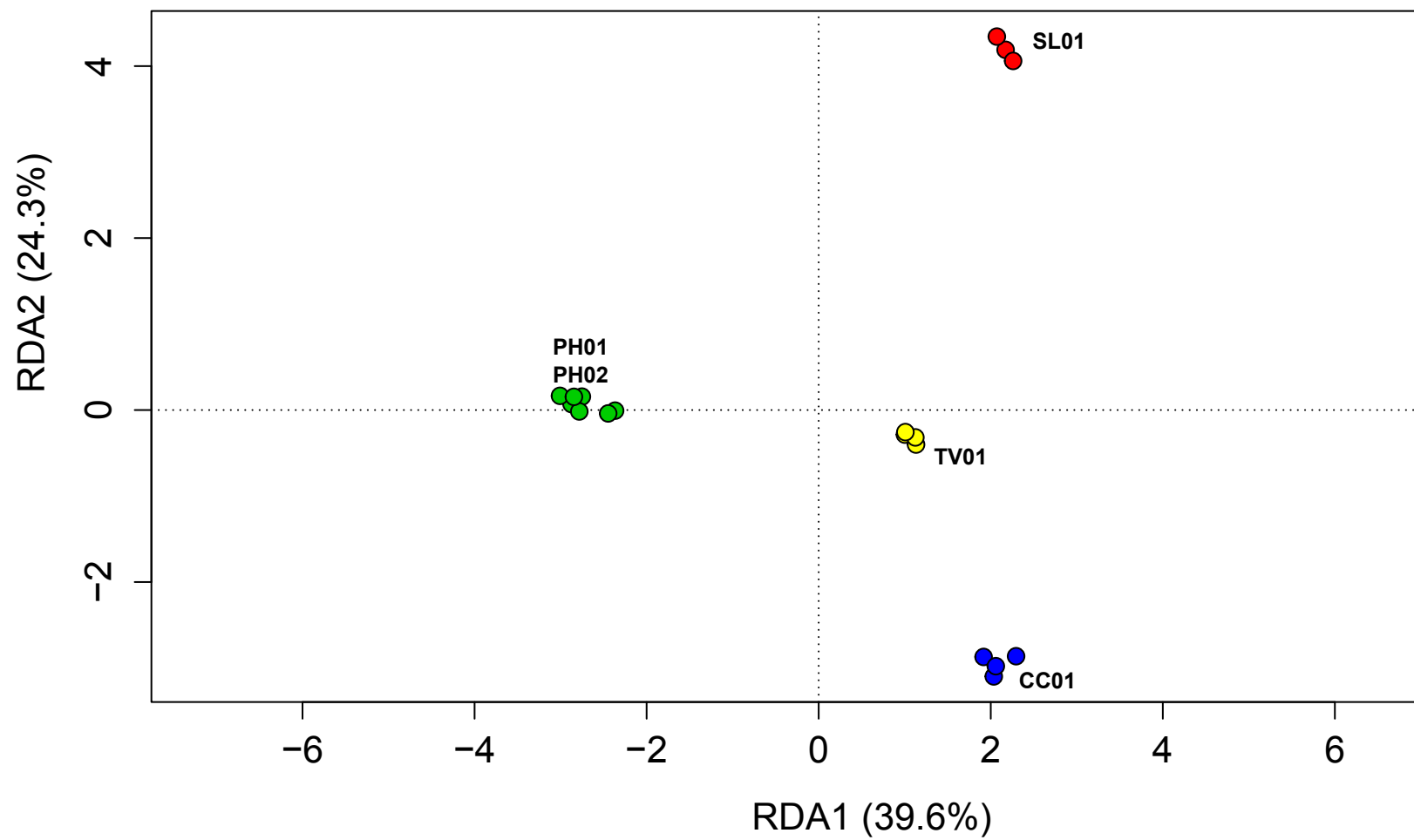

Supplement: Additional file 4: Figure S3. — Redundancy analysis (RDA) plots of microbial profiles obtained from biological replicates of four termite genera. Differences were significantly less between biological replicates than between genera. [file 40168_2015_67_MOESM4_ESM.pdf]

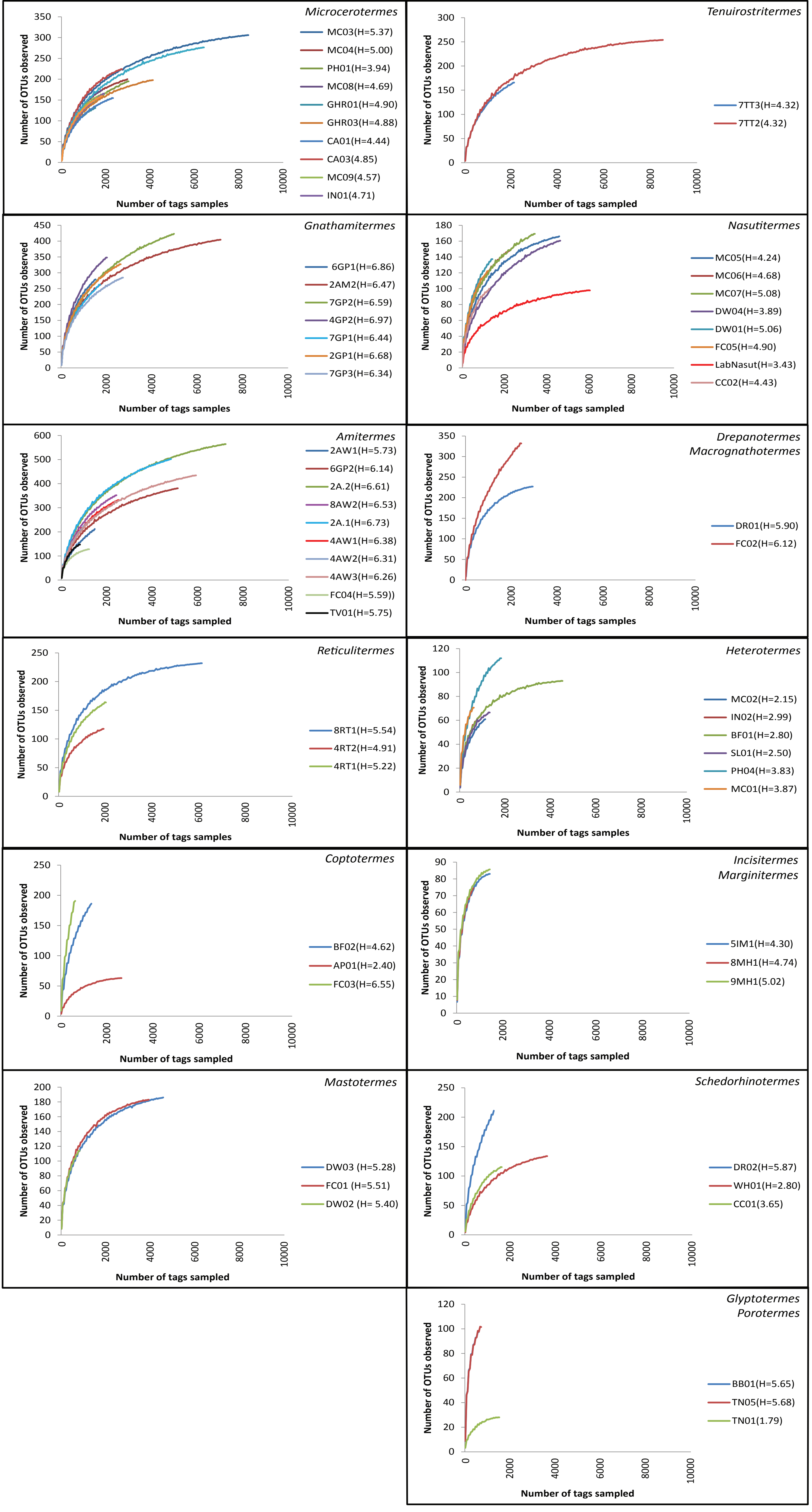

Supplement: Additional file 5: Figure S4. — Rarefaction curves and associated Shannon diversity indices (H) of microbial profiles obtained for each of the 66 samples separated into different panels by termite genus affiliation. [file 40168_2015_67_MOESM5_ESM.pdf]

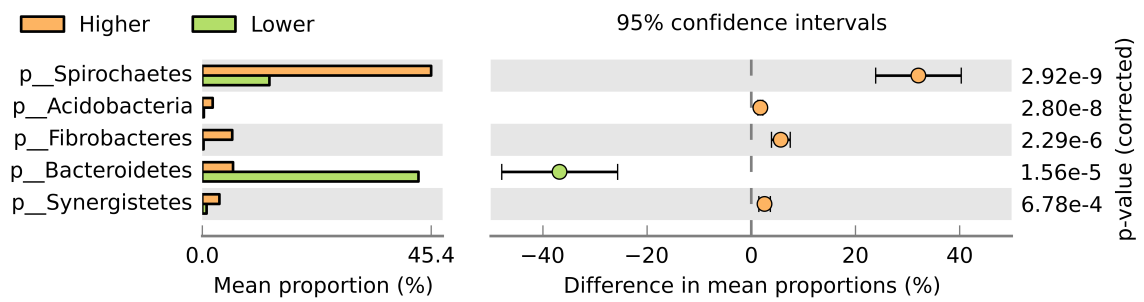

Supplement: Additional file 7: Figure S5. — Core and accessory bacterial phyla with a significant difference in mean proportions ≥1% between higher and lower termites and a p value ≤0.05. Statistical significance was assessed using Welch’s t-test with Šidák multiple test correction. [file 40168_2015_67_MOESM7_ESM.pdf]

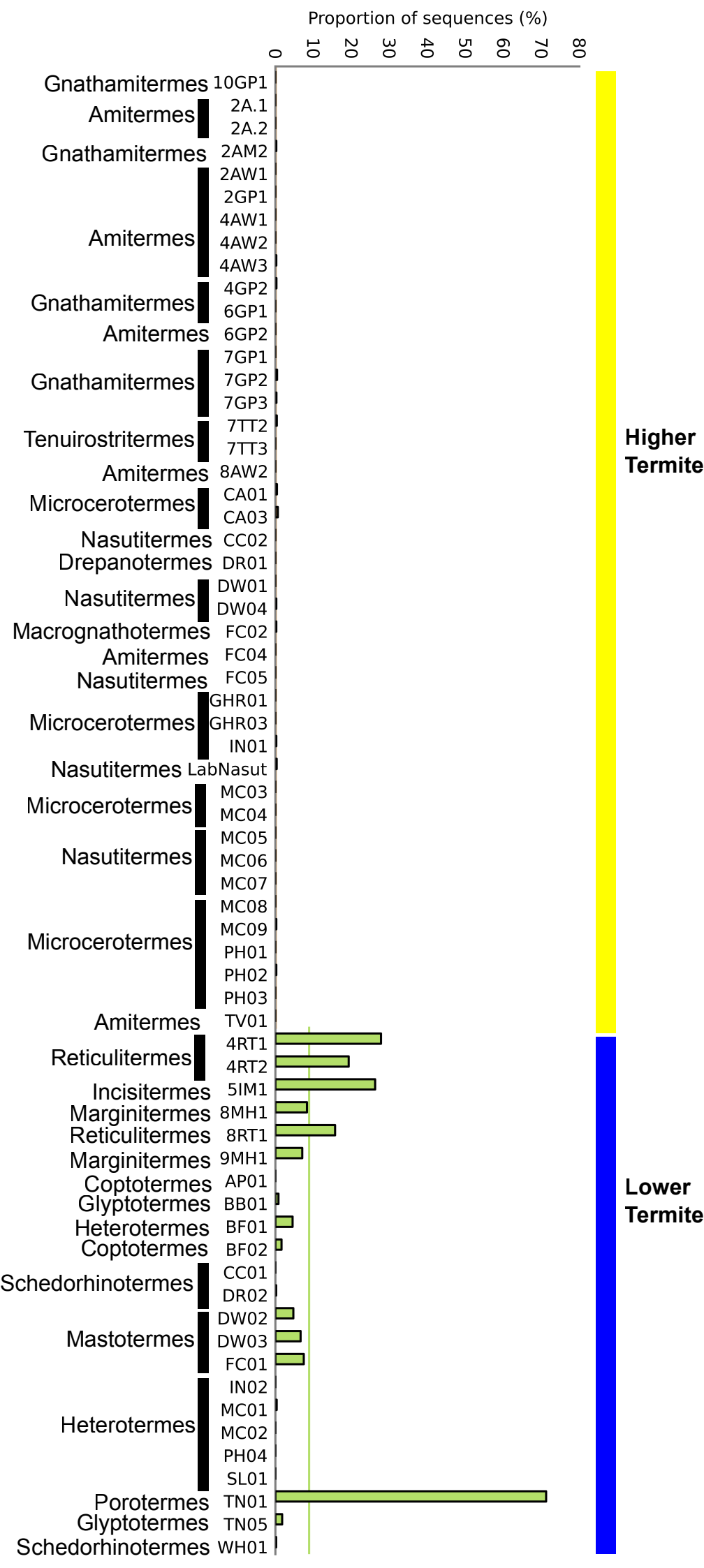

Supplement: Additional file 8: Figure S6. — Relative proportion of Elusimicrobia across the higher and lower termite samples. Termite genus affiliations of the samples is shown to the left of the figure. [file 40168_2015_67_MOESM8_ESM.pdf]

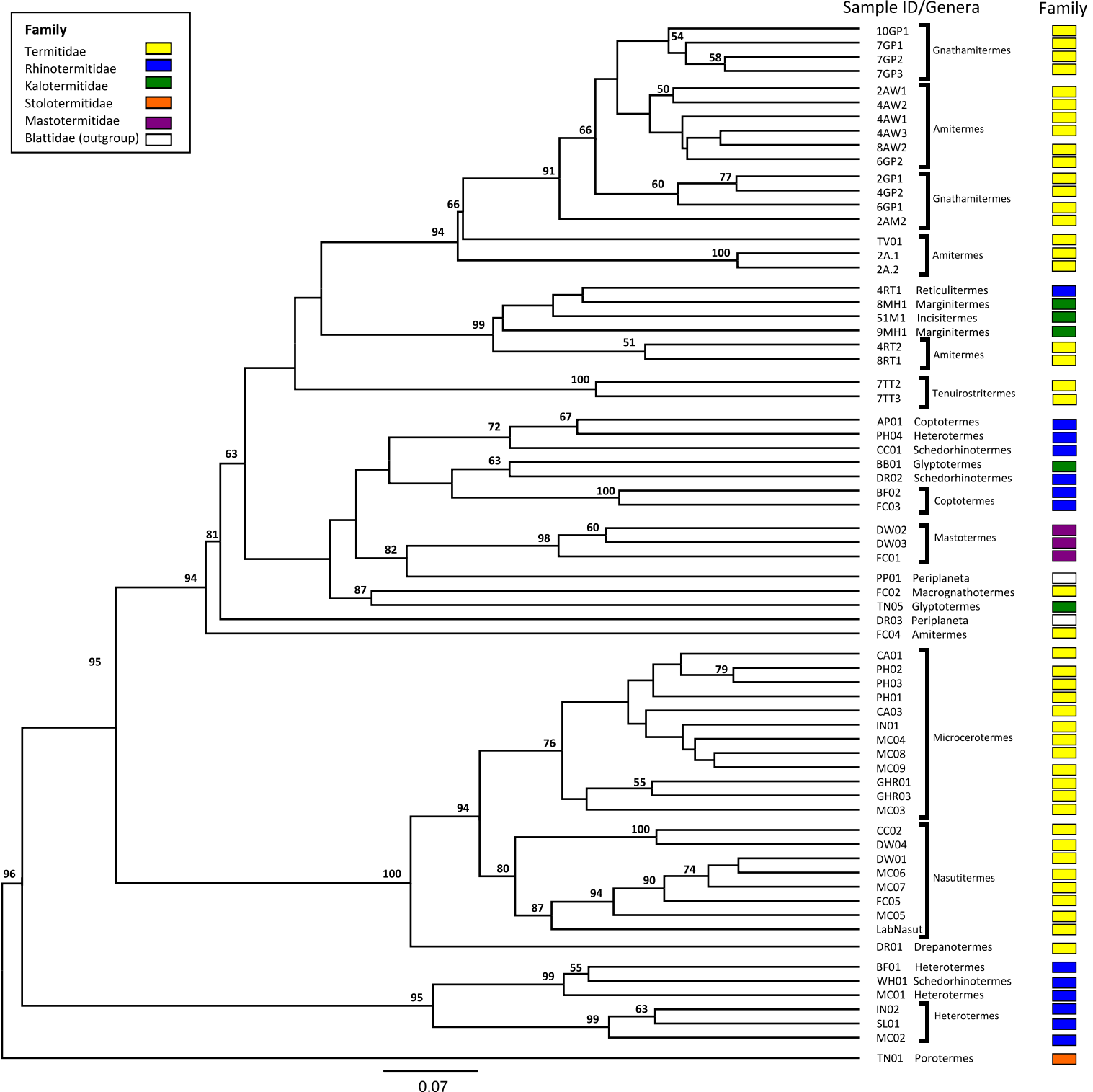

Supplement: Additional file 15: Figure S11. — UPGMA tree of weighted (relative abundance taken into account) Soergel pairwise distances between bacterial profiles showing a drop in consistency with host phylogeny (particularly family level) relative to the unweighted analysis (Figure 4; Additional file 14: Table S4). The values on interior nodes represent jackknife support values ≥49. Termite host affiliation (family) is indicated to the right of the tree. [file 40168_2015_67_MOESM15_ESM.pdf]

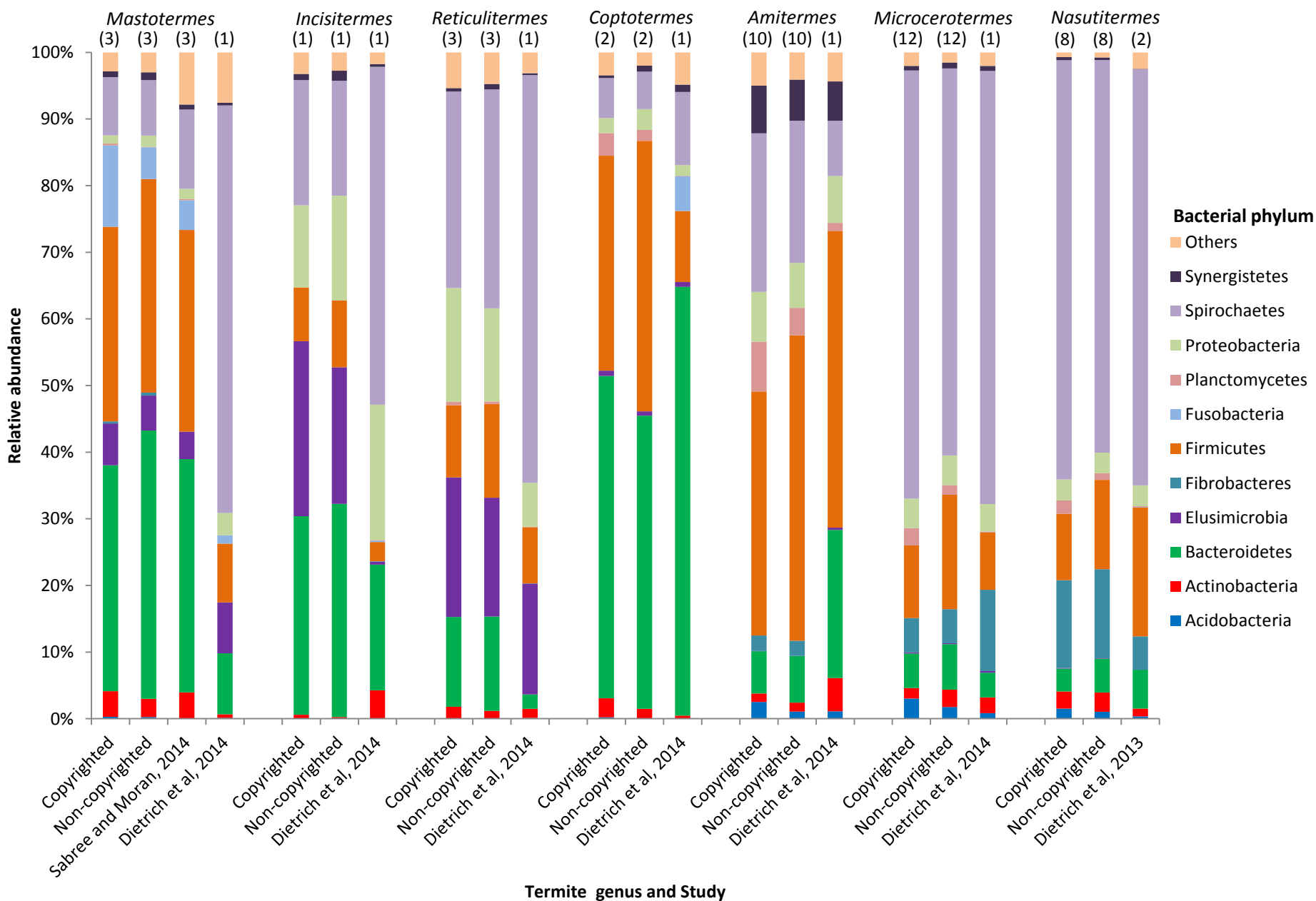

Supplement: Additional file 16: Figure S12. — Comparison of termite gut bacterial profiles obtained in the present study (rRNA copy number corrected and uncorrected profiles) and by [24]. An additional Mastotermes profile reported by [41] is also included for reference. For each study, the profiles are averaged across samples belonging to the same termite genus (number of samples is shown above each bar). [file 40168_2015_67_MOESM16_ESM.pdf]
