# Supplementary material for: A molecular survey of Australian and North American termite genera indicates that vertical inheritance is the primary force shaping termite gut microbiomes
Source: Microbiome. 2015 Feb 25;3:5. doi: 10.1186/s40168-015-0067-8 (PMC4379614; doi:10.1186/s40168-015-0067-8)
Supplement: Additional file 9: Figure S7. — Average whole gut microbial community profiles of the 16 termite genera surveyed in this study. The profiles of the polyphagous termite genera Gnathamitermes and Nasutitermes are further divided by diet (in colored boxes). [file 40168_2015_67_MOESM9_ESM.pdf]

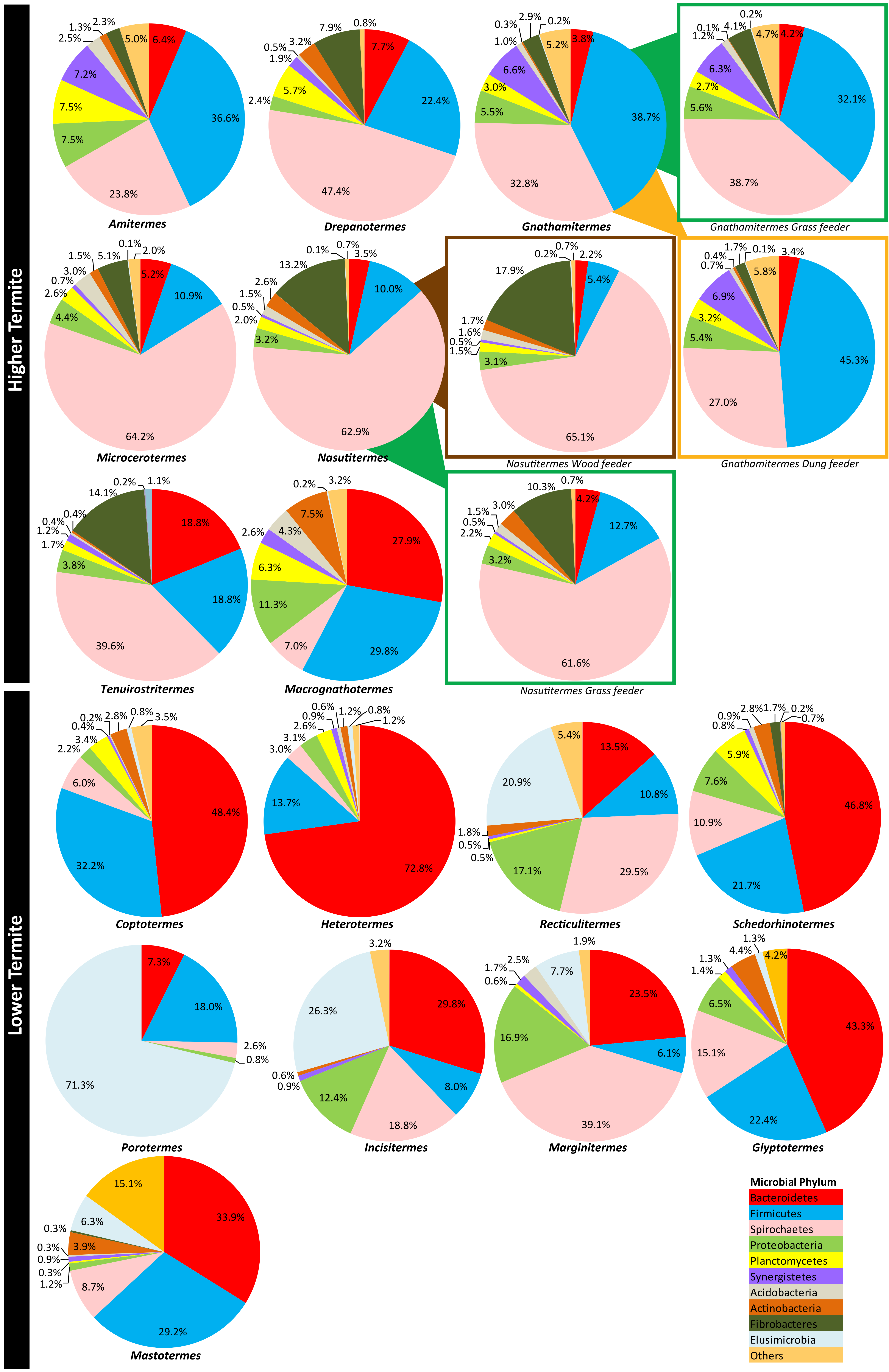
Higher Termite
Lower Termite
Amitermes
Drepanotermes
Gnathamitermes
Gnathamitermes Grass feeder
Microcerotermes
Nasutitermes
Nasutitermes Wood feeder
Gnathamitermes Dung feeder
Tenuirostritermes
Macrognathotermes
Nasutitermes Grass feeder
Coptotermes
Heterotermes
Recticulitermes
Schedorhinotermes
Porotermes
Incisitermes
Marginitermes
Glyptotermes
Mastotermes
Microbial Phylum
Bacteroidetes
Firmicutes
Spirochaetes
Proteobacteria
Planctomycetes
Synergistetes
Acidobacteria
Actinobacteria
Fibrobacteres
Elusimicrobia
Others
