# Supplementary material for: A molecular survey of Australian and North American termite genera indicates that vertical inheritance is the primary force shaping termite gut microbiomes
Source: Microbiome. 2015 Feb 25;3:5. doi: 10.1186/s40168-015-0067-8 (PMC4379614; doi:10.1186/s40168-015-0067-8)
Supplement: Additional file 12: Figure S9. — Heatmap of archaeal OTUs generated with two primer pairs in whole gut samples of termites with ≥10% archaeal relative abundance (Table 2). Each row represents a different OTU, and the abundance as a percentage of the total community is indicated by shading according to the legend. Termite family affiliations of each sample are indicated at the top the figure, respectively, and OTU phylogeny is indicated to the left (phylum) and right (mostly genus) of the figure. [file 40168_2015_67_MOESM12_ESM.pdf]

Relative abundance

- 0.1-2%
- 2-5%
- 5-10%
- 10-25%
- 25-50%
- 50-75%
- >75%

Macrognathotermes		Coptotermes						Schedorhinotermes						Porotermes		Mastotermes						Consensus Lineage	#OTU ID
FC02-803	FC02-927	AP01-803	AP01-926	FC03-803	FC03-926	BF02-803	BF02-926	CC01-803	CC01-926	DR02-803	DR02-926	WH01-803	WH01-926	TN01-803	TN01-926	FC01-803	FC01-926	DW03-803	DW03-926	DW02-803	DW02-926		
9.3	5.9			7.0	15.4	0.2	3.9	0.2		0.2												g__pGrfC24	9
0.2																						g__pGrfC25	1189
0.2				0.2																		g__pGrfC26	1215
				0.2																		g__pGrfC26	1013
0.1																						g__Methanobacteriaceae	1091
				0.1		0.1		0.1								1.1	1.5	0.1	3.3	3.9	4.8	g__Methanobacterium	14
														0.3	0.1							g__Methanobacterium	643
		14.8	18.7			0.3	0.1	28.0	3.3			23.9	23.3	44.1	54.8	1.3	4.5	0.1	4.0	3.1	3.0	g__Methanobrevibacter	2
						0.1	0.1															g__Methanobrevibacter	662
						13.3	21.8	0.1				0.2	0.4									g__Methanobrevibacter	4
								0.2														g__Methanobrevibacter	621
								0.1														g__Methanobrevibacter	1232
								0.1														g__Methanobrevibacter	2226
									0.1			0.1										g__Methanobrevibacter	730
														1.6	1.4							g__Methanobrevibacter	1140
												0.1	0.1									g__Methanobrevibacter	1876
3.1	3.1			3.1	8.2		2.1	1.0	2.3	0.7	0.7	0.3	0.2			0.5	0.9	0.1	1.4	1.9	5.0	g__Methanobrevibacter	23
3.1	3.1			1.4	3.5	0.1	0.9	0.3	0.4			0.1	0.1			2.9	5.6		7.0	3.6	6.1	g__Methanobrevibacter	13
0.7	0.6			0.4	1.5		0.4							0.3	0.5	0.5	0.7		0.1	0.2	1.5	g__Methanobrevibacter	76
0.5	0.2			0.1	0.5		0.1															g__Methanobrevibacter	188
0.1				0.1																		g__Methanobrevibacter	1821
0.1																						g__Methanobrevibacter	1219
	0.1																					g__Methanobrevibacter	1986
				0.1																		g__Methanobrevibacter	1117
								0.1														g__Methanobrevibacter	2468
								0.1														g__Methanobrevibacter	970
								0.1				0.1										g__Methanobrevibacter	2316
										0.1												g__Methanobrevibacter	2447
											0.1											g__Methanobrevibacter	1526
																0.1			0.1	0.1		g__Methanobrevibacter	1199
																0.1	0.1		0.1	0.1		g__Methanobrevibacter	1885
																					0.3	g__Methanobrevibacter	1070
				0.1				0.1														g__Methanocella	2078
				0.1	0.1																	g__Methanospirillum	326
																	0.1	0.1	0.8	0.3	0.1	g__Methanospirillum	97
																				0.1		g__Methanospirillum	1813
0.5	0.6			0.6	1.6		0.4															g__Methanimicrococcus	41
0.1																						g__Methanosarcina	1193
8.7	6.3			5.4	9.1	0.2	2.3	9.3	19.2	0.2	0.1											g__Methanomassiliicoccaceae	10
0.5	0.2			0.2	2.1		0.5															g__Methanomassiliicoccaceae	580
0.2				0.0	0.0																	g__Methanomassiliicoccaceae	1633
0.2									0.2	0.2						0.2	0.5					g__Methanomassiliicoccaceae	247
				0.2																		g__Methanomassiliicoccaceae	2223
								0.2														g__Methanomassiliicoccaceae	913
								0.2														g__Methanomassiliicoccaceae	731
								0.2														g__Methanomassiliicoccaceae	2239
