# Supplementary material for: A molecular survey of Australian and North American termite genera indicates that vertical inheritance is the primary force shaping termite gut microbiomes
Source: Microbiome. 2015 Feb 25;3:5. doi: 10.1186/s40168-015-0067-8 (PMC4379614; doi:10.1186/s40168-015-0067-8)
Supplement: Additional file 13: Figure S10. — Heatmap of protist OTUs (97% seq id) across the 66 termite samples. Each row represents an OTU and each column a gut sample with relative abundance as a percentage of the total microbial community indicated by numbers and shading according to the legend. The termite genus and family for each sample is indicated at the top and bottom of the figure, respectively, and OTU phylogeny is indicated to the left (phylum) and right (mostly genus) of the figure. [file 40168_2015_67_MOESM13_ESM.pdf]

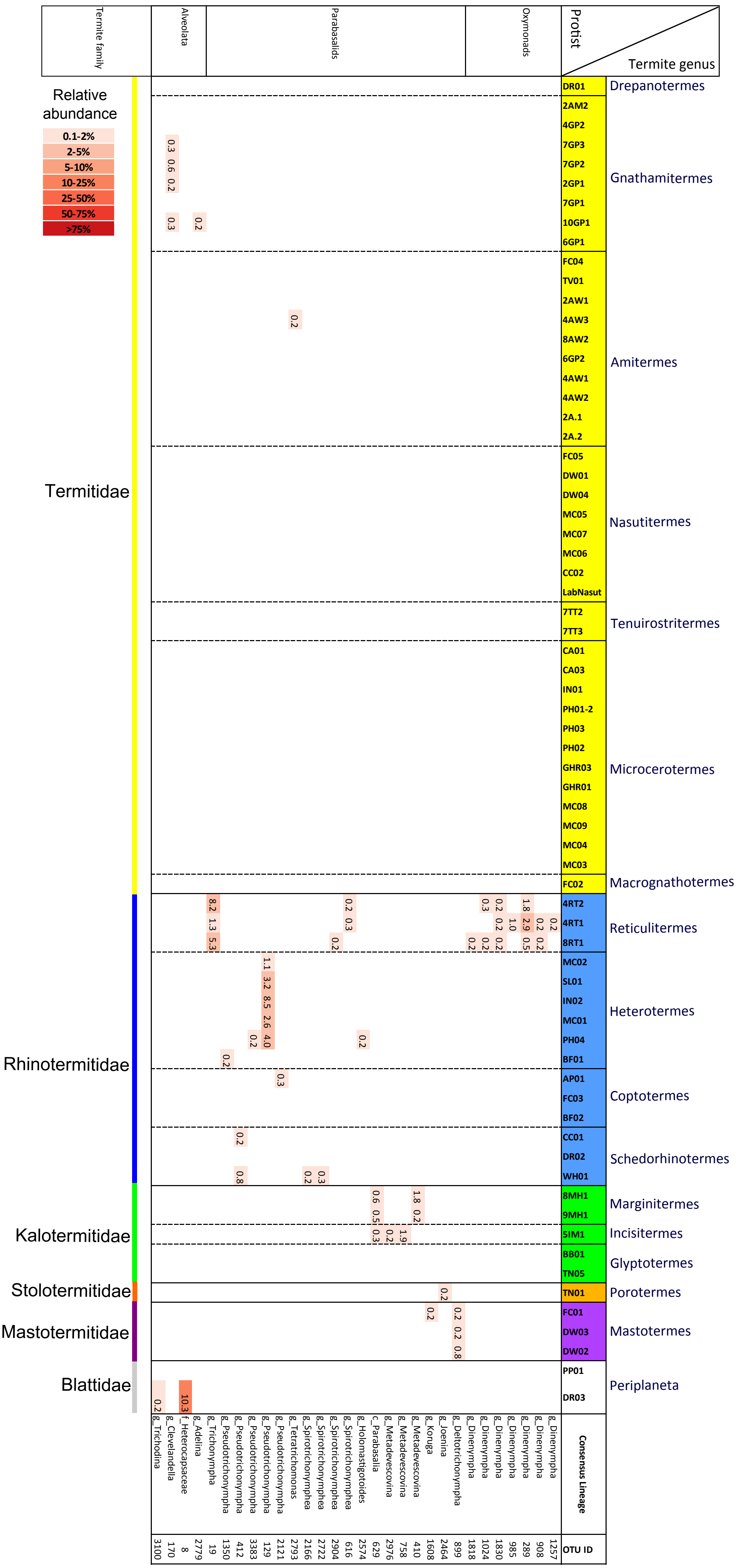

Termite family
Alveolata
Parabasalids
Oxymonads
Protist
Termite genus
Relative abundance
0.1-2%
2-5%
5-10%
10-25%
25-50%
50-75%
>75%
Termitidae
Rhinotermitidae
Kalotermitidae
Stolotermitidae
Mastotermitidae
Blattidae
Drepanotermes
Gnathamitermes
Amitermes
Nasutitermes
Tenuirostritermes
Microcerotermes
Macrognathotermes
Reticulitermes
Heterotermes
Coptotermes
Schedorhinotermes
Marginitermes
Incisitermes
Glyptotermes
Porotermes
Mastotermes
Periplaneta
DR01
2AM2
4GP2
7GP3
7GP2
2GP1
7GP1
10GP1
6GP1
FC04
TV01
2AW1
4AW3
8AW2
6GP2
4AW1
4AW2
2A.1
2A.2
FC05
DW01
DW04
MC05
MC07
MC06
CC02
LabNasut
7TT2
7TT3
CA01
CA03
IN01
PH01-2
PH03
PH02
GHR03
GHR01
MC08
MC09
MC04
MC03
FC02
4RT2
4RT1
8RT1
MC02
SL01
IN02
MC01
PH04
BF01
AP01
FC03
BF02
CC01
DR02
WH01
8MH1
9MH1
5IM1
BB01
TN05
TN01
FC01
DW03
DW02
PP01
DR03
Consensus Lineage
OTU ID
g_Trichodina 3100
g_Clevelandella 170
f_Heterocapsaceae 8
g_Adelina 2779
g_Trichonympha 19
g_Pseudotrichonympha 1350
g_Pseudotrichonympha 412
g_Pseudotrichonympha 3383
g_Pseudotrichonympha 129
g_Pseudotrichonympha 2121
g_Tetratrichomonas 2793
g_Spirotrichonymphea 2166
g_Spirotrichonymphea 2722
g_Spirotrichonymphea 2904
g_Spirotrichonymphea 616
g_Holomastigotoides 2574
c_Parabasalia 629
g_Metadevescovina 2976
g_Metadevescovina 758
g_Metadevescovina 410
g_Koruga 1608
g_Joenina 2464
g_Deltotrichonympha 899
g_Dinenympha 1818
g_Dinenympha 1024
g_Dinenympha 1830
g_Dinenympha 985
g_Dinenympha 289
g_Dinenympha 908
g_Dinenympha 1257
