# Supplementary material for: A molecular survey of Australian and North American termite genera indicates that vertical inheritance is the primary force shaping termite gut microbiomes
Source: Microbiome. 2015 Feb 25;3:5. doi: 10.1186/s40168-015-0067-8 (PMC4379614; doi:10.1186/s40168-015-0067-8)
Supplement: Additional file 10: Figure S8. — Heatmap of bacterial OTUs (97% seq id) with ≥10% relative abundance or ≥50% prevalence across the 66 termite samples (Figure 2). Each row represents an OTU and each column a gut sample with relative abundance as a percentage of the total microbial community indicated by numbers and shading according to the legend. The termite genus and family for each sample is indicated at the top and bottom of the figure, respectively, and OTU phylogeny is indicated to the left (phylum) and right (mostly genus) of the figure. [file 40168_2015_67_MOESM10_ESM.pdf]

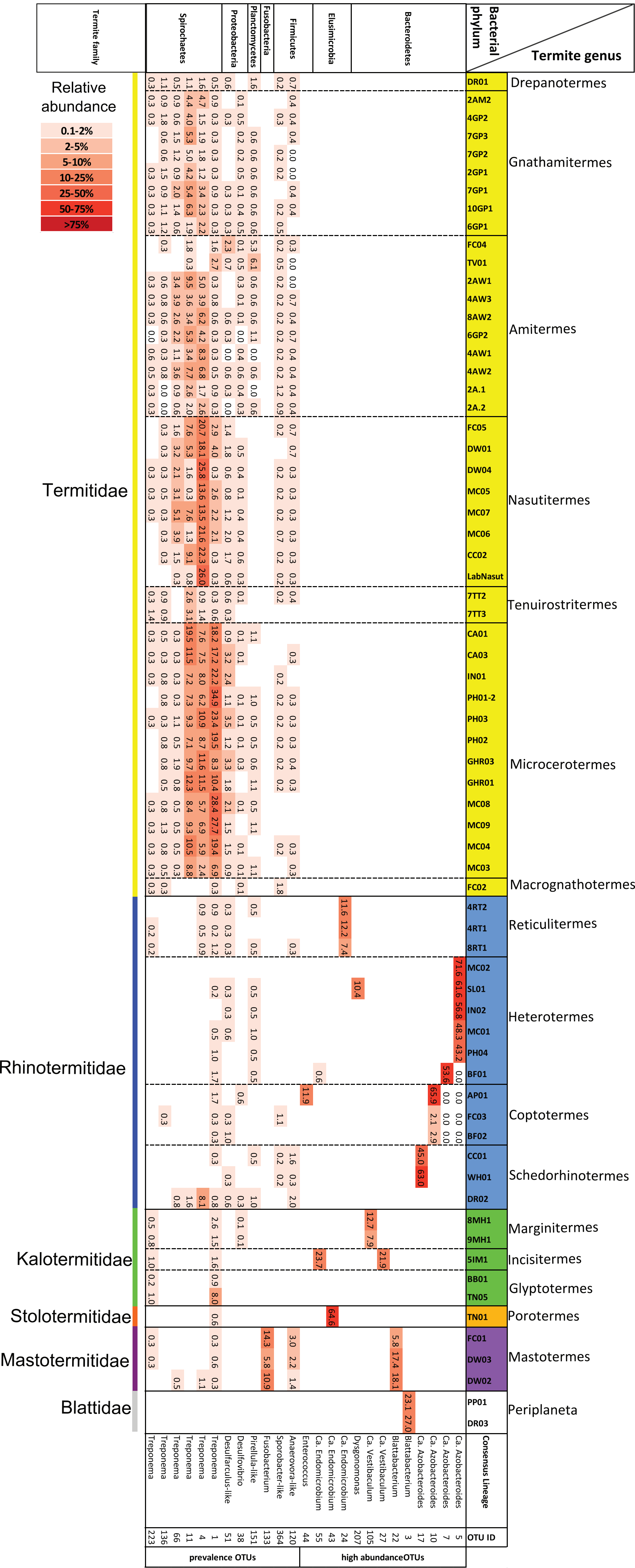
Bacterial phylum
Termite genus
Termite family
Spirochaetes
Proteobacteria
Planctomycetes
Fusobacteria
Firmicutes
Elusimicrobia
Bacteroidetes
Relative abundance
0.1-2%
2-5%
5-10%
10-25%
25-50%
50-75%
>75%
Termitidae
Rhinotermitidae
Kalotermitidae
Stolotermitidae
Mastotermitidae
Blattidae
Drepanotermes
Gnathamitermes
Amitermes
Nasutitermes
Tenuirostritermes
Microcerotermes
Macrognathotermes
Reticulitermes
Heterotermes
Coptotermes
Schedorhinotermes
Marginitermes
Incisitermes
Glyptotermes
Porotermes
Mastotermes
Periplaneta
DR01
2AM2
4GP2
7GP3
7GP2
2GP1
7GP1
10GP1
6GP1
FC04
TV01
2AW1
4AW3
8AW2
6GP2
4AW1
4AW2
2A.1
2A.2
FC05
DW01
DW04
MC05
MC07
MC06
CC02
LabNasut
7TT2
7TT3
CA01
CA03
IN01
PH01-2
PH03
PH02
GHR03
GHR01
MC08
MC09
MC04
MC03
FC02
4RT2
4RT1
8RT1
MC02
SL01
IN02
MC01
PH04
BF01
AP01
FC03
BF02
CC01
WH01
DR02
8MH1
9MH1
5IM1
BB01
TN05
TN01
FC01
DW03
DW02
PP01
DR03
Consensus Lineage
OTU ID
Treponema 223
Treponema 136
Treponema 66
Treponema 11
Treponema 4
Treponema 1
Desulfarculus-like 51
Desulfovibrio 38
Pirellula-like 151
Fusobacterium 133
Sporobacter-like 364
Anaerovorax-like 120
Enterococcus 44
Ca. Endomicrobium 55
Ca. Endomicrobium 43
Ca. Endomicrobium 24
Dysgonomonas 207
Ca. Vestibaculum 105
Ca. Vestibaculum 27
Blattabacterium 22
Blattabacterium 3
Ca. Azobacteroides 17
Ca. Azobacteroides 10
Ca. Azobacteroides 7
Ca. Azobacteroides 5
prevalence OTUs
high abundance OTUs
